# The human gut microbiome in early-onset type 1 diabetes from the TEDDY study

**DOI:** 10.1038/s41586-018-0620-2

**Published:** 2018-10-24

**Authors:** Tommi Vatanen, Eric A. Franzosa, Randall Schwager, Surya Tripathi, Timothy D. Arthur, Kendra Vehik, Åke Lernmark, William A. Hagopian, Marian J. Rewers, Jin-Xiong She, Jorma Toppari, Anette-G. Ziegler, Beena Akolkar, Jeffrey P. Krischer, Christopher J. Stewart, Nadim J. Ajami, Joseph F. Petrosino, Dirk Gevers, Harri Lähdesmäki, Hera Vlamakis, Curtis Huttenhower, Ramnik J. Xavier

**Affiliations:** 1 Broad Institute of MIT and Harvard, Cambridge, MA USA; 2 Department of Biostatistics, Harvard T. H. Chan School of Public Health, Boston, MA USA; 3 Health Informatics Institute, Morsani College of Medicine, University of South Florida, Tampa, FL USA; 4 Department of Clinical Sciences, Lund University/CRC, Skåne University Hospital SUS, Malmo, Sweden; 5 Pacific Northwest Research Institute, Seattle, WA USA; 6 Barbara Davis Center for Childhood Diabetes, University of Colorado, Aurora, CO USA; 7 Center for Biotechnology and Genomic Medicine, Medical College of Georgia, Augusta University, Augusta, GA USA; 8 Department of Pediatrics, Turku University Hospital, Turku, Finland; 9 Institute of Biomedicine, Research Centre for Integrative Physiology and Pharmacology, University of Turku, Turku, Finland; 10 Institute of Diabetes Research, Helmholtz Zentrum München, Munich, Germany; 11 Forschergruppe Diabetes, Technische Universität München, Klinikum Rechts der Isar, Munich, Germany; 12 Forschergruppe Diabetes e.V. at Helmholtz Zentrum München, Munich, Germany; 13 National Institute of Diabetes & Digestive & Kidney Diseases, Bethesda, MD USA; 14 Alkek Center for Metagenomics and Microbiome Research, Department of Molecular Virology and Microbiology, Baylor College of Medicine, Houston, TX USA; 15 Institute of Cellular Medicine, Newcastle University, Newcastle upon Tyne, UK; 16 Department of Computer Science, Aalto University, Espoo, Finland; 17 Gastrointestinal Unit, Center for the Study of Inflammatory Bowel Disease, and Center for Computational and Integrative Biology, Massachusetts General Hospital and Harvard Medical School, Boston, MA USA; 18 Center for Microbiome Informatics and Therapeutics, MIT, Cambridge, MA USA; 19 Present Address: Janssen Human Microbiome Institute, Janssen Research and Development, Cambridge, MA USA

**Keywords:** Paediatric research, Metagenomics, Microbiome, Type 1 diabetes

## Abstract

Type 1 diabetes (T1D) is an autoimmune disease that targets pancreatic islet beta cells and incorporates genetic and environmental factors^1^, including complex genetic elements^2^, patient exposures^3^ and the gut microbiome^4^. Viral infections^5^ and broader gut dysbioses^6^ have been identified as potential causes or contributing factors; however, human studies have not yet identified microbial compositional or functional triggers that are predictive of islet autoimmunity or T1D. Here we analyse 10,913 metagenomes in stool samples from 783 mostly white, non-Hispanic children. The samples were collected monthly from three months of age until the clinical end point (islet autoimmunity or T1D) in the The Environmental Determinants of Diabetes in the Young (TEDDY) study, to characterize the natural history of the early gut microbiome in connection to islet autoimmunity, T1D diagnosis, and other common early life events such as antibiotic treatments and probiotics. The microbiomes of control children contained more genes that were related to fermentation and the biosynthesis of short-chain fatty acids, but these were not consistently associated with particular taxa across geographically diverse clinical centres, suggesting that microbial factors associated with T1D are taxonomically diffuse but functionally more coherent. When we investigated the broader establishment and development of the infant microbiome, both taxonomic and functional profiles were dynamic and highly individualized, and dominated in the first year of life by one of three largely exclusive *Bifidobacterium* species (*B. bifidum*, *B. breve* or *B. longum*) or by the phylum Proteobacteria. In particular, the strain-specific carriage of genes for the utilization of human milk oligosaccharide within a subset of *B. longum* was present specifically in breast-fed infants. These analyses of TEDDY gut metagenomes provide, to our knowledge, the largest and most detailed longitudinal functional profile of the developing gut microbiome in relation to islet autoimmunity, T1D and other early childhood events. Together with existing evidence from human cohorts^7,8^ and a T1D mouse model^9^, these data support the protective effects of short-chain fatty acids in early-onset human T1D.

## Abstract (web summary)

An analysis of more than 10,000 metagenomes from the TEDDY study provides a detailed functional profile of the gut microbiome in relation to islet autoimmunity, and supports the protective effects of short-chain fatty acids in early-onset type 1 diabetes.

## Main

Recent literature has linked several facets of gut health with the onset of T1D in humans and rodent models^4,6,10^. Altered intestinal microbiota in connection to T1D has been reported in Finnish^7,8,11,12^, German^13^, Italian^14^, Mexican^15^, American (Colorado)^16^ and Turkish^17^ children. Common findings include increased numbers of *Bacteroides* species, and deficiency of bacteria that produce short-chain fatty acids (SCFAs)^7,8^ in cases of T1D or islet autoimmunity (IA)^8,11,15,18^. Corroborating these findings, decreased levels of SCFA-producing bacteria were found in adults with type 2 diabetes (T2D)^19^. In addition, increased intestinal permeability^14^ and decreased microbial diversity^12^ after IA but before T1D diagnosis have been reported. Studies using the nonobese diabetic (NOD) mouse model have determined immune mechanisms that mediate the protective effects of SCFAs^9^ and the microbiome-linked sex bias in autoimmunity^20^. NOD mice fed specialized diets resulting in high bacterial release of the SCFAs acetate and butyrate were almost completely protected from T1D^9^. A study in a streptozotocin-induced T1D mouse model demonstrated that bacterial products recognized in pancreatic lymph nodes contribute to pathogenesis^21^.

Even in the absence of immune perturbation, the first few weeks, months and years of life represent a unique human microbial environment that has only recently been detailed^22,23^. Infants have a markedly different gut microbial profile from adults, characterized by a distinct taxonomic profile, greater proportion of aerobic energy harvest metabolism, and more extreme dynamic change^24^. These differences gradually fade over the first few years of life, particularly in response to the introduction of solid food, and individual microbial developmental trajectories are influenced by environment, delivery mode, breast (versus formula) feeding, and antibiotics^25–27^. Most studies that address the development of the gut microbiome, both generally and in association with T1D, have used gene analysis of 16S rRNA, which leaves open the question of functional and strain-specific differences that are not easily detected by this technology that might contribute to disease pathogenesis^12^.

Bridging this gap is one goal of the The Environmental Determinants of Diabetes in the Young (TEDDY) study, a prospective study that aims to identify environmental causes of T1D^28^. It includes six clinical research centres in the United States (Colorado, Georgia/Florida and Washington) and Europe (Finland, Germany and Sweden), which together have recruited several thousand newborns with a genetic predisposition for T1D or first-degree relative(s) with T1D. This has enabled the TEDDY study to collect a range of biospecimens, including monthly stool samples starting at three months of age, coupled with extensive clinical and personal data such as diet, illnesses, medications and other life experiences. To characterize microbial, environmental, genetic, immunological and additional contributors to the development of T1D, the TEDDY study group further assembled nested case–control studies for IA (*n* = 418 case–control pairs) and T1D (*n* = 114)^29^. Case–control pairs were matched by clinical centre, sex and family history of T1D, which are all known confounding factors for T1D susceptibility and microbiome composition.

**Fig. 1:**
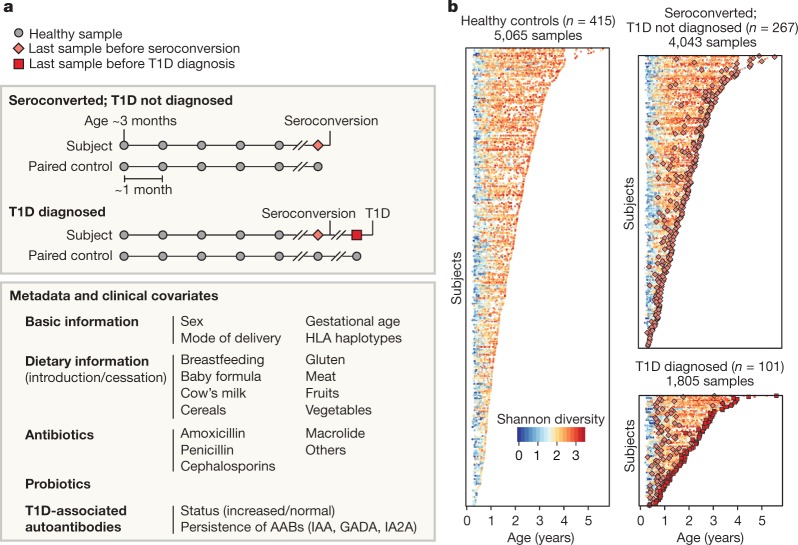
More than 10,000 longitudinal gut metagenomes from the TEDDY T1D cohort. We analysed 10,913 metagenomes collected longitudinally from 783 children (415 controls, 267 seroconverters, and 101 diagnosed with T1D) approximately monthly over the first five years of life. **a**, Subjects were recruited at six clinical centres (Finland, Sweden, Germany, Washington, Georgia and Colorado). Primary end points were seroconversion (defined as persistent confirmed IA) and T1D diagnosis. Additional metadata analysed for subjects and samples included the status of breastfeeding, birth mode, probiotics, antibiotics, formula feeding, and other dietary covariates. **b**, Overview of stool samples collected and microbiome development as summarized by Shannon’s alpha diversity and stratified by end point. Median number of samples per individual *n* = 12 (healthy controls *n* = 10, seroconverters *n* = 13, T1D cases *n* = 16).

Here, we assessed 783 children followed from three months to up to five years of age from six clinical centres in four countries (Finland, Germany, Sweden and the United States) who either progressed to persistent IA or T1D or were matched as controls (Fig. 1a, b, Extended Data Table 1). Stool samples were collected, on average, monthly starting at three months of age and continuing until the clinical end point (IA or T1D). This study focused solely on analysing metagenomic sequencing data (*n* = 10,903 samples, *n* = 783 subjects), while a companion paper by Stewart et al.^30^ interrogated corresponding 16S rRNA amplicon sequencing information.

**Fig. 2:**
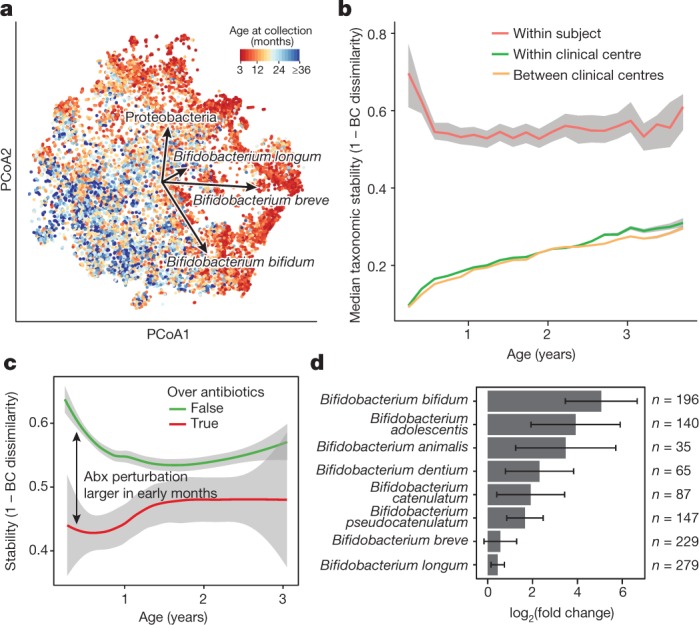
The early gut microbiome is characterized by early heterogeneity of *Bifidobacterium* species and individualized accrual of taxa over time. **a**, Principal coordinate analysis (PCoA) ordination of microbial beta diversities (*n* = 10,913 samples), measured by Bray–Curtis dissimilarity. Arrows show the weighted averages of key taxonomic groups. **b**, Microbiota stability, measured by Bray–Curtis (BC) dissimilarity (*n* = 10,750 samples) in three-month time windows, over two-month increments, stratified into three groups: within subject, within clinical centre, and between clinical centres. Lines show median values per time window. Shaded area denotes the estimated 99% confidence interval. Gut microbial communities were highly individual. **c**, Influence of antibiotic (Abx) courses on microbial stability, measured by Bray–Curtis dissimilarity over consecutive stool samples (<50 days apart) from the same individual during the first three years of life, and stratified by whether antibiotics were given between the two samples (*n* = 654 observations with antibiotics, *n* = 6,734 observations without antibiotics). Curves show locally weighted scatterplot smoothing (LOESS) for the data per category. Shaded areas show permutation-based 95% confidence intervals for the fit. **d**, Decreases in the most common *Bifidobacterium* species in connection to oral antibiotic treatments. Fold change was measured between consecutive samples with an antibiotic course between them, given that the species in question was present in the first of the two samples. Sample size per species (*n*) indicates the number of sample pairs in which the species in question was present in the sample before the antibiotic treatment. Bars show bootstrapped mean log_2_(fold change) (that is, decrease), and error bars denote s.d. (*n* = 1,000 bootstrap samples).

**Extended Data Fig. 1:**
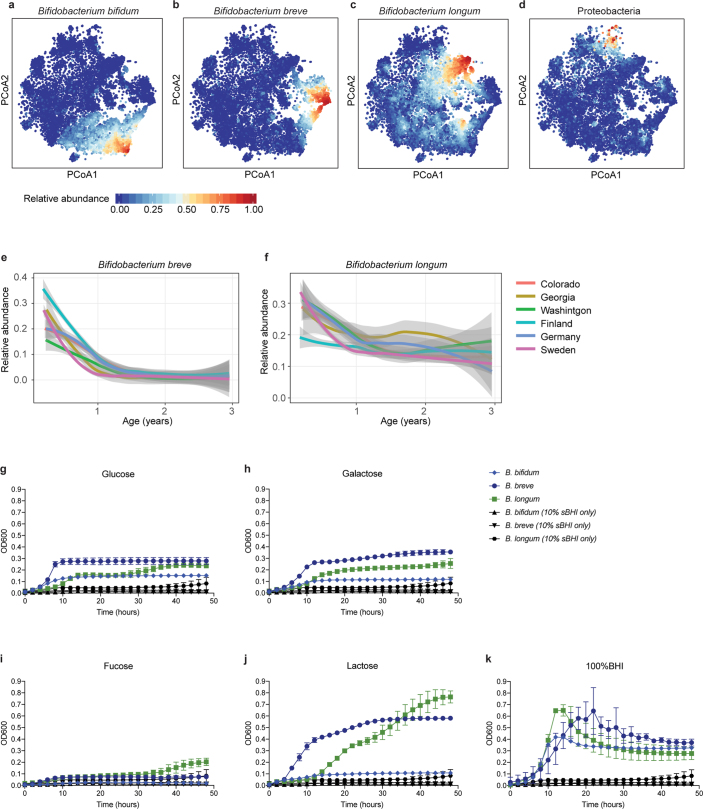
Heterogeneity in early taxonomic profiles. **a**–**d**, Relative abundances of taxonomic groups highlighted by weighted averages in Fig. 2a (arrows) shown separately (*n* = 10,913 samples). **e**, **f**, Average longitudinal abundance of *B. breve* (**e**) and *B. longum* (**f**) per clinical centre (*n* = 10,194 samples). The curves show LOESS fits for the relative abundances, and shaded area shows 95% confidence interval for each fit, as implemented in geom_smooth function in ggplot2 R package. **g**–**k**, Growth curves of human infant isolates of *B. breve*, *B. bifidum* and *B. longum* grown individually in low-nutrient medium (10% sBHI) supplemented with single carbon sources (glucose (**g**), galactose (**h**), fucose (**i**) and lactose (**j**)) or grown in 100% sBHI (**k**). As a negative control, growth curves of each strain grown in 10% BHI without additional sugar are shown in black for each condition. Data are representative of three independent experiments and are presented as the mean and s.d. of triplicate assessments.

**Extended Data Fig. 2:**
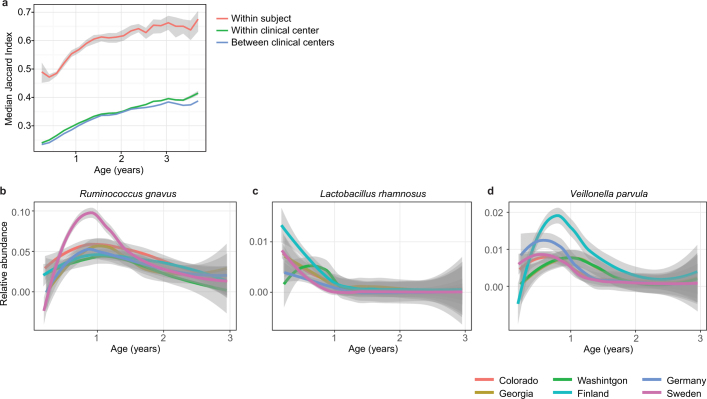
Stability and regional differences of taxonomic profiles. **a**, Stability of the microbiota, measured by the Jaccard index (*n* = 10,750 samples) in three-month time windows, over two-month increments, stratified into three groups: within subject, within clinical centre, and across clinical centres. Lines show the median per time window. Shaded areas show the 99% confidence interval estimated using binomial distribution. Compare to Fig. 2b, which shows the same analysis measured by Bray–Curtis dissimilarity. **b**–**d**, Average longitudinal abundance of *Ruminococcus gnavus* (**b**), *Lactobacillus rhamnosus* (**c**) and *Veillonella parvula* (**d**) per clinical centre (*n* = 10,194 samples). The curves show LOESS fit for the relative abundances, as above.

**Extended Data Fig. 3:**
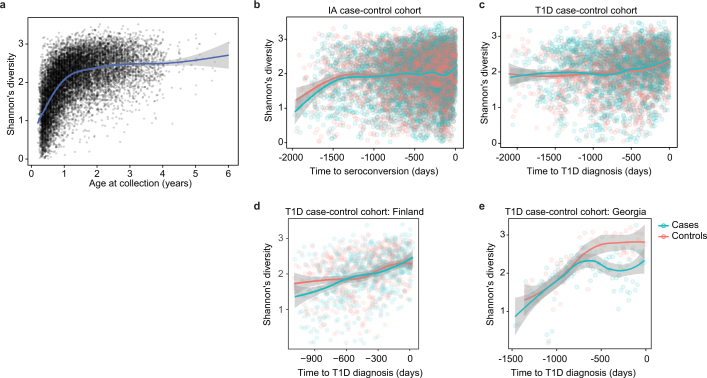
Accrual of microbial alpha diversity. **a**, Shannon’s diversity of the taxonomic profiles of the gut microbial communities (*n* = 10,913 samples) with respect to the age at the sample collection. The curve shows the generalized additive model (GAM) fit for the data, and the shaded area shows the 95% confidence interval for each fit, as implemented in geom_smooth function in ggplot2 R package. **b**, Shannon’s diversity for the samples in the IA case–control cohort (*n* = 7,051) with respect to the time to the appearance of first autoantibody (seroconversion). The curves show LOESS fits for cases and controls separately, and the shaded area shows 95% confidence intervals for each fit. **c**, Shannon’s diversity for the samples in the T1D case–control cohort (*n* = 3,309) with respect to the time to T1D diagnosis. The curves and shaded areas are as in **b**. **d**, As in **c**, but only for data (*n* = 983 samples) for subjects in Finland. No difference between cases and controls. **e**, As in **c**, but only for data (*n* = 142 samples, *n* = 6 subjects) for subjects in Georgia, USA. Cases show a drop in alpha diversity before the diagnosis of T1D (linear mixed-effects model, *P* = 0.0033).

We first investigated the taxonomic composition of early gut metagenomes at the species level. Principal coordinate analysis ordination of Bray–Curtis beta diversities showed a strong longitudinal gradient and marked heterogeneity among the earliest samples (Fig. 2a, Extended Data Fig. 1a–k, Supplementary Note 1). Permutational analysis of variance (ANOVA) of Bray–Curtis beta diversities indicated that inter-subject differences explained 35% of microbial taxonomic variation (permutation test, *P* < 0.001, 1,000 permutations), followed by age at stool sampling at roughly 4% of variance (*P* < 0.001). Using cross-sectional analysis to test for associations between taxonomic beta diversities and other collected metadata, we found that in addition to subject ID and age, geographical location and breastfeeding had strong and systematic effects on the composition of the microbial community (Supplementary Table 1, Extended Data Fig. 2a–d, Supplementary Note 1). To investigate the stability and individuality of the microbial profiles further, we compared intra- and inter-subject Bray–Curtis beta diversities. The gap between individual stability and similarity within or across clinical centres was largest at the beginning of the sampling period, indicating that the children had particularly dissimilar microbiota during these early months (Fig. 2b, Supplementary Note 1). Finally, we tested microbial alpha diversity (Shannon’s diversity index) of taxonomic profiles for associations with collected metadata, and found that the cessation of breastfeeding had the largest effect (ANOVA, partial *η*^2^ = 0.053) in the accrual of alpha diversity in early life (Supplementary Table 2, Extended Data Fig. 3a–e, Supplementary Note 1).

**Extended Data Fig. 4:**
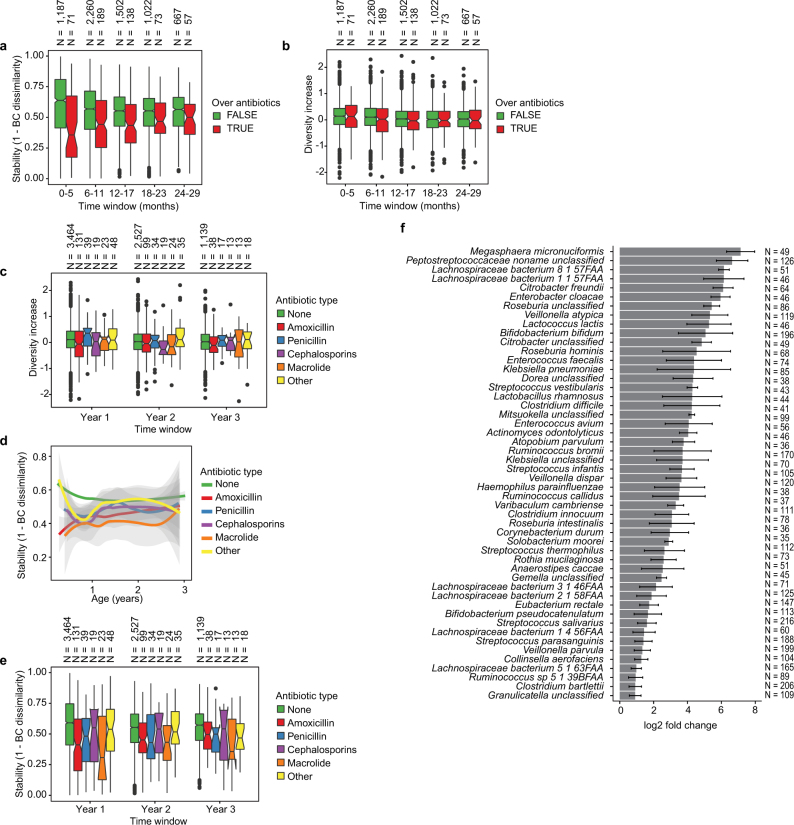
Effects of antibiotics. **a**, Influence of antibiotic courses on microbial stability, stratified into six-month time windows (*x* axis). Stability was measured by Bray–Curtis dissimilarity over consecutive stool samples (<50 days apart) from the same individual between 3 and 29 months of age, and stratified by whether antibiotics were given between the two samples. For each notched box plot, the box denote the interquartile range (IQR), the horizontal line denotes the median, and the notch denotes the approximation for the 95% confidence interval (notch width = 1.58 × IQR/*n*^0.5^, in which *n* is the number of samples per box plot). Compare to Fig. 2c. **b**, Influence of antibiotic courses on microbial diversity. Notched box plots denote the increase (difference) in diversity between two consecutive stool samples (<50 days apart) stratified by antibiotic administration between the samples. Data show no difference between the groups (antibiotics versus no antibiotics). **c**, Influence of antibiotics courses on microbial diversity by antibiotic type; data from **b** stratified into one-year time windows (*x* axis) and antibiotic types. No significant differences were detected between the antibiotic types. **d**, **e**, Influence of antibiotic courses on microbial stability by antibiotic type; data from Fig. 2c and Extended Data Fig. 3a stratified by antibiotic type. **d**, LOESS fit for the relative abundances (shaded area shows 95% confidence interval for each fit, as implemented in geom_smooth function in ggplot2 R package). **e**, Notched box plots (as in **a** and **b**) for the data per antibiotic type. No significant differences were detected between the antibiotic types. No antibiotics, *n* = 7,130; amoxicillin, *n* = 268; penicillin, *n* = 90; cephalosporin, *n* = 51; macrolide, *n* = 60; other, *n* = 101. **f**, Decreases in relative abundance of bacteria over antibiotic courses. Bacteria for which the bootstrapped 95% confidence interval of the fold change does not overlap zero are shown. Fold change was measured between consecutive samples with an antibiotic course between them, given that the species in question was present in the first of the two samples. Sample size per species (*n*) indicate the number of sample pairs in which the species in question was present in the sample preceding the antibiotic treatment. Bars denote bootstrapped mean log_2_(fold change) (that is, decrease), and error bars denote s.d. (*n* = 1,000 bootstrap samples).

We next investigated the effects of antibiotics on the early life microbiome. Courses of oral antibiotics disrupted microbial stability, with a larger effect in the earliest comparisons (Fig. 2c, Extended Data Fig. 4a–f, Extended Data Table 2, Supplementary Note 2). Previous studies have found *Bifidobacterium* species to be especially vulnerable to antibiotics^31,32^, leading us to investigate how antibiotic perturbations influenced these common dominant members of the early gut. Comparing microbial relative abundances before and after antibiotics (assuming that the given species was present in the preceding sample), we saw a decrease in the abundances of the *Bifidobacterium* members *B. bifidum*, *B. pseudocatenulatum*, *B. adolescentis*, *B. dentium* and *B. catenulatum*, whereas *B. longum* and *B. breve* did not systematically decline owing to antibiotics (Fig. 2d), suggesting that certain *Bifidobacterium* species are particularly susceptible to out-competition by other community members after depletion by antibiotics. Given their dominance in the typical developing gut microbiota and finely tuned balance of metabolic interactions with breast milk, this finding underscores the importance of approaching antibiotic prescriptions in early childhood with care, especially during breastfeeding.

**Extended Data Fig. 5:**
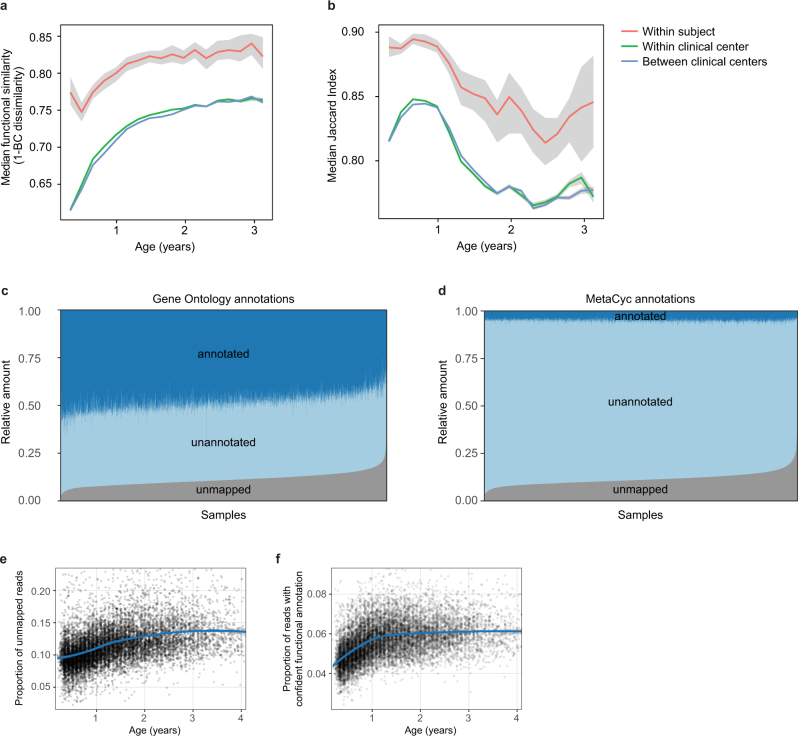
Dynamics of species-specific microbial functional potential during early gut development. **a**, **b**, Stability of microbial pathways (*n* = 10,580 samples) measured by Bray–Curtis dissimilarity (**a**) and the Jaccard index (**b**) and stratified into three groups: within subject, within clinical centre, and across clinical centres. Although the baseline level of functional similarity is significantly greater than that of taxa (see Fig. 2b), functional states and development trajectories also both retain a level of personalization. The stability of the functional profiles was evaluated in three-month time windows, over two-month increments. Lines show the median per time window, and shaded area denotes the 99% confidence interval estimated using binomial distribution. **c**, **d**, Proportion of metagenomic gene abundance with functional annotation through Gene Ontology (**c**) and MetaCyc (**d**) databases. The metagenomic reads were divided into the following categories: reads that could be mapped to genes with functional assignment in the database in question (annotated), and reads with no annotation but alignment to species pangenomes or UniProt proteins (unannotated). The proportion of the unknown genes (unmapped) was estimated using the number of reads with unknown origin. **e**, The proportion of unmapped reads, reflecting the relative abundances of reads not mappable to any microbial pangenomes in the available reference set or to UniProt. An increasing trend of unmapped reads with respect to the age at sample collection continued through approximately two years of age. **f**, The proportion of reads with confident functional annotation in MetaCyc within the genes that mapped to species pangenomes or UniProt proteins. The data again showed an increasing longitudinal trend, implicating a deficit of functional and biochemical annotations within microorganisms that are abundant during the first year of life.

Accompanying our taxonomic profiling, functional profiling of these metagenomes suggested the development of a consistent microbial functional core during infancy, with a smaller subject-specific variable functional pool (Extended Data Fig. 5a, b, Supplementary Note 3). As in most microbial community studies^33^, microbial gene families of uncharacterized function made up a substantial fraction of these profiles, averaging roughly 50% based on Gene Ontology^34^ annotations (Extended Data Fig. 5c) and more than 90%  based on more functionally specific MetaCyc pathways (Extended Data Fig. 5d). We observed an increasing longitudinal trend in the proportion of unmapped reads (Extended Data Fig. 5e, Pearson’s *r* = 0.318, *P* < 2.2 × 10^−16^). However, within the reads that mapped to either microbial pangenomes or known protein sequences (the proportion of which decreased with age), we saw an increase in the proportion of reads with MetaCyc annotation, mainly during the first year (Extended Data Fig. 5f, Pearson *r* = 0.391, *P* < 2.2 × 10^−16^). This suggests that although the early life microbiome is relatively well-covered by current microbial reference genomes, less functional and biochemical characterization has been carried out on gene families within these microorganisms, which will thus particularly benefit from future work.

**Fig. 3:**
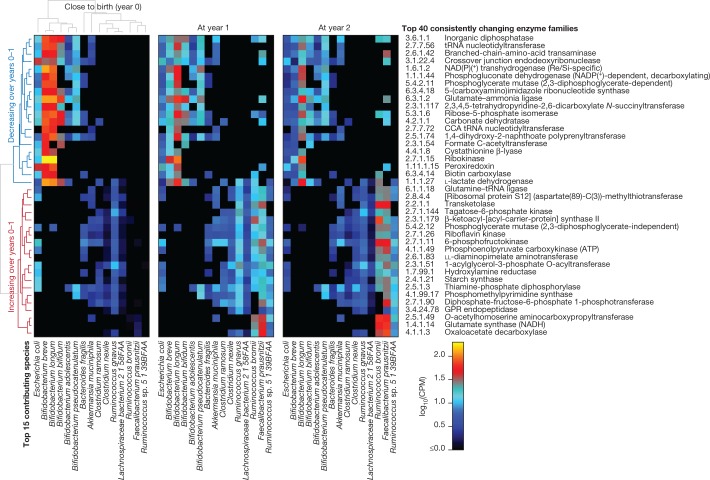
Consistent changes in enzymatic content of the gut microbiome in early life. We identified enzyme families (level-4 Enzyme Commission (EC) categories) that exhibited the most consistent within-subject changes in total community abundance between the ages of 3 months and 1 year. The top 20 most consistent increases or decreases are presented and stratified according to their top 15 contributing species. Heat map values reflect the mean contribution of each species to each enzyme over samples (*n* = 733 at 3 months; 675 at 1 year; and 382 at 2 years). Values reflect units of copies per million (CPM) normalized to total read depth (including unmapped reads and reads mapped to gene families lacking EC annotation). Rows (enzymes) and columns (species) are clustered according to Spearman correlation at 3 months; subsequent years are ordered according to clustering at 3 months.

In addition to broadly conserved and subject-specific functions, we identified a range of microbial metabolic enzymes that consistently increased or decreased in abundance over the first year of life, paralleling shifts in community structure and infant diet (Fig. 3, Supplementary Note 3, Supplementary Table 3). For example, the enzyme l-lactate dehydrogenase (1.1.1.27), which is well-characterized in Bifidobacteria for its role in milk fermentation^35^, was among the most consistently declining enzymes over this period, notably coinciding with the cessation of breastfeeding in many infants (from 73% breastfed at month 3 to 28% at year 1). Conversely, the enzyme transketolase (2.2.1.1), which has been implicated previously^36^ in the metabolism of fibre, was among the most consistently increasing enzymes, which also coincided with increased incorporation of solid food (a component of 53% of infants’ diets at month 3 versus 100% at year 1). Hence, these notable changes in community functional potential highlight the unique metabolic environment of the early infant gut, and the subsequent transition to a more adult-like gut microbiome that is adapted to variable, fermentative energy sources.

**Extended Data Fig. 6:**
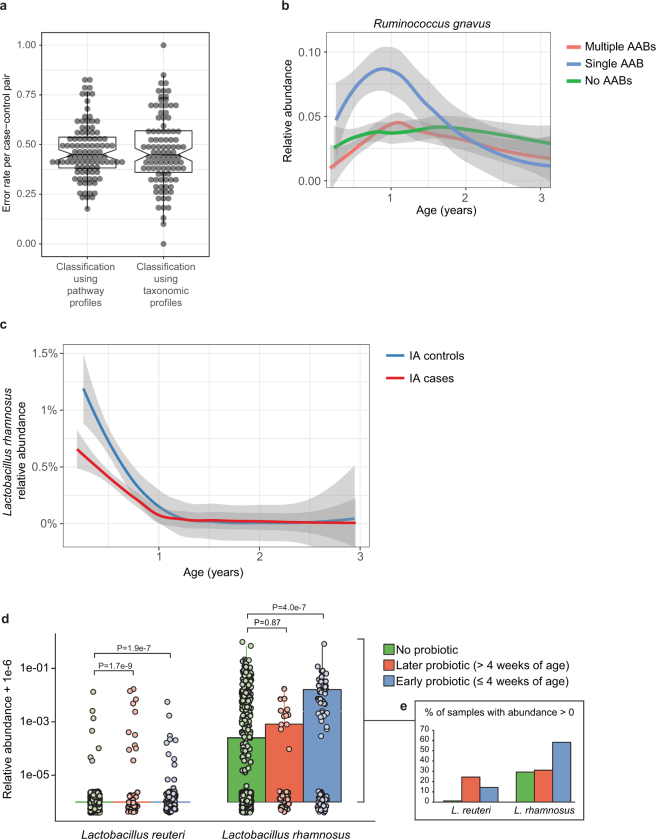
Differences between cases and controls. **a**, The gut microbiome functional (left) and taxonomic (right) profiles were classified between cases and controls using leave-one-out cross-validation (*n* = 3,366 samples), in which one case–control pair was held-out in turn. Data show error rates for classifying these held-out samples per fold (a data point per fold, *n* = 100 folds). This suggests weak but better-than-random classification between cases and controls. Notched box plots are as in Extended Data Fig. 4. **b**, Average longitudinal abundance of *Ruminococcus gnavus* in Finland (*n* = 2,630 samples) stratified by the number of observed persistent autoantibodies (AABs); no autoantibodies (that is, healthy control), a single autoantibody, or multiple (two or more) autoantibodies. **c**, Average longitudinal abundance of *Lactobacillus rhamnosus* in IA cases and controls (*n* = 7,017 samples). *L. rhamnosus* is more abundant in controls (*q* = 0.055). The curves in **b** and **c** show LOESS fit per group, and shaded areas show 95% confidence interval for each fit, as implemented in geom_smooth function in ggplot2 R package. **d**, Abundance (left) and prevalence (right) of *Lactobacillus reuteri* and *L. rhamnosus* in the first stool sample of each individual (collected at approximately three months of age) in association with early probiotic supplementation. ‘No probiotic’ indicates no probiotics given before the first stool sample (*n* = 583); ‘later probiotic’ refers to probiotics given later than the first four weeks but before the first stool sample (*n* = 45); ‘early probiotic’ refers to probiotics given during the first four weeks of life (*n* = 84). Numbers (*n*) per clinical centre are given in Extended Data Table 2. *L. reuteri* and *L. rhamnosus* were more abundant and prevalent in groups with probiotics supplementation. Visual jitter was added to make data equal to zero distinguishable, and boxes denote the IQR, when applicable. The shown *P* values were obtained by applying Fisher’s exact test (two-sided) to presence or absence count data (counting samples in which the species were present).

Combining taxonomic and functional profiles to test for differences between cases and controls, we used linear mixed-effects modelling and identified a relatively small number of individual taxonomic and functional features that were associated with case–control outcome (Supplementary Table 4), most with borderline statistical significance (false discovery rate (FDR) corrected *q*-values indicated below). We confirmed separation between cases and controls by random forest classifiers (Extended Data Fig. 6a, b, Supplementary Note 4). In the IA case–control cohort, healthy controls contained higher levels of *Lactobacillus rhamnosus* (*q* = 0.055), supporting protection against IA by early probiotic supplementation^37^ (Extended Data Fig. 6c, d, Supplementary Note 5). IA controls also had more *Bifidobacterium dentium* (*q* = 0.054), whereas IA cases had on average higher abundance of *Streptococcus* group *mitis/oralis/pneumoniae* species (*q* = 0.11). In T1D case–control comparisons, controls had higher levels of *Streptococcus thermophilus* (*q* = 0.078) and *Lactococcus lactis* (*q* = 0.094) species, both common in dairy products, whereas cases contained higher levels of species such as *Bifidobacterium pseudocatenulatum* (*q* = 0.078), *Roseburia hominis* (*q* = 0.11) and *Alistipes shahii* (*q* = 0.14). Even though our modelling approach controlled for regional differences in clinical centres, we found additional but often weak associations with outcome in some clinical centres when tested separately (Supplementary Table 4). Finnish IA cases had more *Streptococcus* group *mitis/oralis/pneumoniae* species (*q* = 0.0008), IA controls from Colorado had more *Streptococcus thermophilus* (*q* = 0.0059), and Swedish IA cases contained more *Bacteroides vulgatus* (*q* = 0.090).

Pathways with the highest statistical significance in case–control comparisons were related to bacterial fermentation (Supplementary Table 4). The superpathway of fermentation (MetaCyc identifier PWY4LZ-257) was increased in controls in the T1D cohort (*q* = 0.019) and Finnish IA cohort (*q* = 0.049). SCFAs such as butyrate, acetate and propionate are common by-products of bacterial fermentation, and butyrate and acetate protected NOD mice against T1D^9^. Consistently, we observed that several bacterial pathways that contribute to the biosynthesis of short-chain fatty acids were increased in healthy controls. Among pathways involved in butyrate production, the degradation of l-arginine, putrescine and 4-aminobutanoate (ARGDEG-PWY) superpathway was increased in T1D controls cohort-wide (*q* = 0.043), whereas the fermentation of acetyl coenzyme A to butanoate (PWY-5676) was more abundant in the Finnish T1D controls (*q* = 0.053). The degradation of acetylene (P161-PWY), which contributes to acetate production, was increased in T1D controls cohort-wide (*q* = 0.14), and the degradation of l-1,2-propanediol (PWY-7013), which is involved in propionate biosynthesis, was higher in the German T1D controls (*q* = 0.019). These findings support existing evidence for the protective effects of SCFAs in human T1D^7,8^ and T2D^19^ cohorts and the NOD mouse model^9^.

As reflected by the community-level analyses, human milk with its pro- and prebiotic functions is one of the main factors that determine the community composition of the infant gut microbiome. *Bifidobacterium longum* subsp. *infantis* is a particularly versatile degrader of human milk oligosaccharide (HMO) that is often found in stool samples collected during breastfeeding^38^. By following the families representing genes in the *B. longum* subsp. *infantis* HMO gene cluster^39,40^ in our data, we found that an additional 30 bacterial species carried at least one homologue with more than 50% sequence identity to one or more HMO utilization genes (Supplementary Table 5). As expected, many Bifidobacteria carried several homologues, but surprisingly three *Enterococcus* species (*E. casseliflavus*, *E. faecalis* and *E. faecium*) also carried seven or more homologues (Supplementary Table 5).

**Fig. 4:**
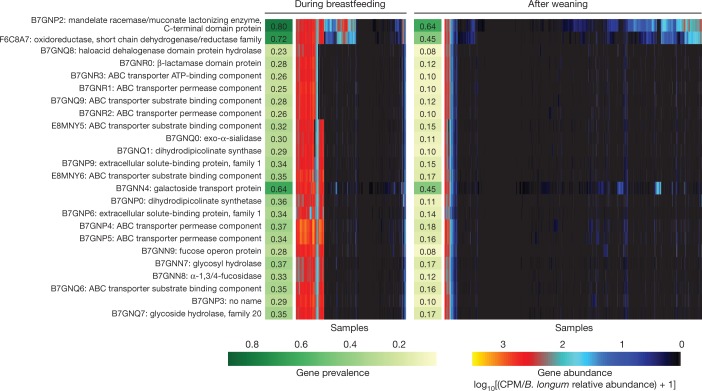
*Bifidobacterium longum* strains are characterized by HMO gene content and stratified by breastfeeding status. Gene families involved in HMO utilization and showing contrasting presence in *B. longum* genomes during breastfeeding (*n* = 1,584 samples) compared to after weaning (*n* = 3,705 samples). Abundance heat map columns represent stool samples in which the relative abundance of *B. longum* species was more than 10% (*n* = 5,289 samples). Rows and columns were ordered by hierarchical clustering using the complete linkage method. As in Fig. 3, values reflect units of CPM and were further divided by relative abundance of *B. longum* to obtain quantifications that are comparable between samples. UniRef90 identifiers and gene names or families are indicated on the left.

**Extended Data Fig. 7:**
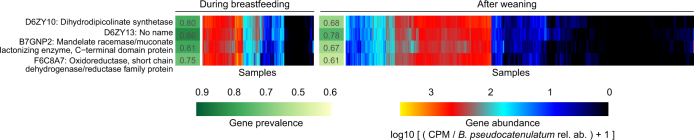
Contrasting HMO utilization genes in *B. pseudocatenulatum*. The gene families involved in HMO utilization and that show contrasting presence in *B. pseudocatenulatum* genomes during breastfeeding (*n* = 321 samples) compared to after weaning (*n* = 1,004 samples). Columns represent stool samples in which the relative abundance of *B. pseudocatenulatum* species was greater than 10% (*n* = 1,325 samples). Rows and columns were ordered by hierarchical clustering using complete linkage method. Compare to Fig. 4, which shows similar data for *B. longum*. UniRef90 identifiers and gene names or families are indicated on the left.

To identify strain-level adaptation similar to *B. longum* subsp. *infantis*, we further examined whether any of these genes showed contrasting prevalence between samples collected during breastfeeding and after weaning, given that the carrier species itself was present. In total, 41 gene families were observed more often during breastfeeding (Supplementary Table 5, test of proportions, adjusted *P* < 0.001); most (37 out of 41) were carried by *B. longum* (Fig. 4), and *B. pseudocatenulatum* contained four such gene families (Extended Data Fig. 7, Supplementary Table 5). In samples with *B. longum*, this implicated a clear strain shift after weaning, when fewer *B. longum* strains carried these genes (Fig. 4). In samples with *B. pseudocatenulatum*, four gene families showed a similar but less contrasting pattern (Extended Data Fig. 7). Overall, these observations identify new candidate species that contribute to HMO processing or exploitation, and link strain composition to specific driving molecular functions that potentially explain selective sweeps during microbiome development, in this case specifically related to breastfeeding.

Despite ample sample size, scrutiny of the study design, and thorough statistical analyses, most of the taxonomic and functional signals we detected in case–control comparisons were modest in effect size and statistical significance. This could be due to several reasons—differences between T1D endotypes, temporally diffuse signals, geographical heterogeneity, or lack of stool samples for the first two months of life —and these should be considered in future investigations (Supplementary Note 6). Furthermore, the data used in these investigations was composed of samples from the genetically predisposed and mostly white, non-Hispanic case–control groups designed into the TEDDY study. Results cannot be guaranteed to reflect the whole TEDDY cohort or child populations in the respective countries.

Future targeted approaches to identify subject-specific connections between the gut microbiota and T1D pathogenesis may be beneficial, particularly given the apparent population-level heterogeneity revealed here. For example, laboratory experiments involving dietary factors that have been associated with the onset of T1D^3^ may reveal biochemically specific signals that are mediated by the microbiome. Different endotypes of disease, such as differences in the first appearing autoantibody (IAA versus GADA), the number of appearing autoantibodies, the time from seroconversion to T1D diagnosis, genetic host risk alleles and ethnic backgrounds, may be characterized by distinct microbial configurations (Supplementary Note 6). Finally, components of the microbiome that were poorly measured in these data may also have crucial roles: viruses, fungi, microbial transcription or small-molecule biochemistry. By surveying these additional molecular activities by cross-sectional analysis and in more detailed longitudinal populations, this study lays the foundation to identify further gut microbial components that are predictive, protective or potentially causal in T1D risk or pathogenesis.

## Methods

### Cohort and study design

TEDDY is a prospective cohort study funded by the National Institutes of Health with the primary goal to identify environmental causes of T1D. It includes six clinical research centres—three in the United States (Colorado, Georgia/Florida, Washington) and three in Europe (Finland, Germany and Sweden). Detailed study design and methods have been previously published^28,41,42^. Written informed consents were obtained for all study participants from a parent or primary caretaker, separately, for genetic screening and participation in a prospective follow-up. The TEDDY study was approved by local US Institutional Review Boards and European Ethics Committee Boards in Colorado’s Colorado Multiple Institutional Review Board, Georgia’s Medical College of Georgia Human Assurance Committee (2004–2010), Georgia Health Sciences University Human Assurance Committee (2011–2012), Georgia Regents University Institutional Review Board (2013–2015), Augusta University Institutional Review Board (2015–present), Florida’s University of Florida Health Center Institutional Review Board, Washington state’s Washington State Institutional Review Board (2004–2012) and Western Institutional Review Board (2013–present), Finland’s Ethics Committee of the Hospital District of Southwest Finland, Germany’s Bayerischen Landesärztekammer (Bavarian Medical Association) Ethics Committee, Sweden’s Regional Ethics Board in Lund, Section 2 (2004–2012) and Lund University Committee for Continuing Ethical Review (2013–present). The study is monitored by External Advisory Board formed by the National Institutes of Health.

This analysis used stool samples and clinical metadata from two nested case–control studies (persistent, confirmed IA or T1D) using risk set sampling^29^. The data used here were collected as of 31 May 2012, as a 1:1 match in which one control per case of persistent confirmed IA or T1D was selected from the full TEDDY cohort. A control was a participant who had not developed persistent, confirmed IA or T1D by the time the case to which it was matched had developed IA or T1D, within ±45 days of the event time. Matching factors were clinical centre, sex and family history of T1D to control for differences in geographical area, genetic background and in sample or data handling between clinical centres. In all case–control comparisons, we removed all case–control pairs in which the control later progressed to case status (that is, progressed to IA or T1D). In additional, 17 subjects with missing information about breastfeeding together with their matched pairs were excluded from the case–control comparisons to avoid confounding effects from unknown breastfeeding status.

The development of persistent, confirmed IA was assessed every three months. Persistent autoimmunity was defined by the presence of confirmed islet autoantibody on two or more consecutive visits. The date of persistent autoimmunity was defined as the draw date of the first sample of the two consecutive samples that deemed the child persistently positive for a specific autoantibody (or any autoantibody). T1D was defined according to American Diabetes Association criteria for diagnosis^43^.

Stool samples were collected monthly starting at three months of age and continuing up until 48 months of age, then every three months until 10 years of age and then biannually thereafter, into the three plastic stool containers provided by the clinical centre. Children who were antibody negative after 4 years of age were encouraged to submit four times a year even though after 4 years their visits schedule switched to biannual. Parents sent the stool containers at either ambient or +4 °C temperature with guaranteed delivery within 24 h in the appropriate shipping box to the NIDDK repository if living in the United States or their affiliated clinical centre if living in Europe. The European clinical centres stored the stool samples and sent monthly bulk shipments of frozen stool to the NIDDK repository. The TEDDY Manual of Operations, including the stool sample collection protocol, can be accessed online at https://repository.niddk.nih.gov/static/studies/teddy/teddy_moop.pdf.

A priori power calculations using discrete Cox’s proportional hazards regression^44^ for the matched IA case–control study estimated 80% power, *α* = 0.01, two-sided test to detect an odds ratio > 3 for an exposure with 5% prevalence, to an odds ratio > 1.8 for an exposure with 20% prevalence. The experiments were not randomized, and investigators were not blinded to allocation during experiments and outcome assessment.

### Metagenomic sequencing and initial bioinformatics

Samples were metagenomically sequenced as one library each multiplexed through Illumina HiSeq machines using the 2 × 100-bp paired-end read protocol. Samples with limited DNA quantity and/or too few high-quality reads were filtered out, resulting in a discrepancy of sample frequencies between the metagenomic data and the 16S rRNA amplicon sequencing data analysed in the companion paper^30^. Casava v1.8.2 (Illumina) output initial FASTQ files from the resulting data were processed using cutadapt v1.9dev2 for adaptor removal, Trim Galore v0.2.8 (Babraham Bioinformatics) for removing low-quality bases and PRINSEQ v0.20.3^45^ for sample demultiplexing. Bowtie2 v2.2.3 was used to map reads to the human genome for decontamination before subsequent analysis.

### Taxonomic and functional profiling by MetaPhlAn2 and HUMAnN2

Taxonomic profiling of the metagenomic samples was performed using MetaPhlAn2^46^ v2.6.0, which uses a library of clade-specific markers to provide pan-microbial (bacterial, archaeal, viral and eukaryotic) quantification at the species level. MetaPhlAn2 was run using default settings.

Functional profiling was performed with HUMAnN2^47^ v0.9.4. For an input metagenome, HUMAnN2 constructs a sample-specific reference database by concatenating and indexing the pangenomes of species detected in the sample by MetaPhlAn2 (pangenomes are pre-clustered, pre-annotated catalogues of open reading frames found across isolate genomes from a given species^48^). HUMAnN2 then maps sample reads against this database to quantify gene presence and abundance in a species-stratified manner, with unmapped reads further used in a translated search against UniRef90^49^ to include taxonomically unclassified but functionally distinct gene family abundances. Finally, for community-total, species-stratified, and unclassified gene family abundance, HUMAnN2 reconstructs metabolic pathway abundance based on the subset of gene families annotated to metabolic reactions (based on reaction and pathway definitions from MetaCyc^50^). Enzyme (level-4 Enzyme Commission (EC) categories) abundances were further computed by summing the abundances of individual gene families annotated to each EC number based on UniRef90-EC annotations from UniProt^51^.

### Phenotype and covariate analysis

This study includes extensive collection of clinical covariates that cover several aspects of common and rare life events in early childhood from infancy up to five years of age. In these analyses, we used information that is, according to the literature, of high relevance in terms of gut microbiome development. Information about mothers, pregnancy and birth was collected during the three-month clinic visit by questionnaire and included the mode of birth (vaginal birth versus caesarean section), gestational age, infant’s 5-min Apgar score, information about maternal diabetes (T1D, T2D or gestational diabetes) and maternal insulin and medication use (antibiotics, angiotensin-converting enzyme inhibitors, metformin, glyburide, antihypertensives) during pregnancy. Dietary information used in these analyses includes the start (and end) date for the following dietary compounds: breastfeeding, baby formula, cow’s milk, gluten, cereals, meat, vegetables and fruits. The start of solid food (anything other than breast milk or cow’s milk) was also analysed separately. The T1D-associated autoantibodies IAA, GADA and IA2A were analysed from serum samples collected at each clinic visit. In addition to IA, defined as persistent, confirmed autoantibody seropositivity, we analysed the data in terms of the persistency and cumulative frequency of autoantibodies (single or multiple autoantibodies). In TEDDY, all prescribed antibiotic courses are recorded. We further stratified these data by the type of antibiotic in five categories: amoxicillin, penicillin, cephalosporins, macrolide and other antibiotics. Information about probiotics covered the dates for starting and stopping probiotic supplementation, but not the specific types of probiotics used. In addition, sex, information about whether first degree relatives in family had T1D, and HLA haplotypes of the subjects were used in these analyses. Subjects screened from the general population were identified with high-risk alleles (89%) including: DRB1*04-DQA1*03-DQB1*03:02/DRB1*03-DQA1*05-DQB1*02:01 (DR3/4), DRB1*04-DQA1*03-DQB1*03:02/DRB1*04-DQA1*03-DQB1*03:02 (DR4/4), DRB1*04-DQA1*03-DQB1*03:02/DRB1*08-DQA1*04-DQB1*04:02 (DR4/8) and DRB1*03-DQA1*05-DQB1*02:01/DRB1*03-DQA1*05-DQB1*02:01 (DR3/3), plus six genotypes specific to first-degree relatives^28^.

Principal coordinate analysis (PCoA) ordination was generated using *t*-distributed stochastic neighbour embedding (*t*-SNE) as implemented in Rtsne package in R with Bray–Curtis dissimilarity as the distance measure and perplexity (a free parameter) equal to 50. Statistical significance of the trends between early clusters and metadata were tested using mixed-effect logistic regression and samples collected during the first year of life as follows. The target variable used was a binary indicator of whether the relative abundance of the taxon of interest (three different *Bifidobacterium* species or phylum Proteobacteria) was greater than 0.5 (definition of the cluster). The age of sample collection, mode of delivery, clinical centre, breastfeeding status (ongoing/stopped), solid food status (binary variable indicating whether solid food was introduced in the diet) and antibiotics status (binary variable indicating whether the subject received antibiotics during the last 30 days) were used as fixed effects, and the subject ID was used as a random effect.

Associations between microbial feature abundances and clinical outcome were determined using MaAsLin^52^. In brief, this multivariate linear modelling system for microbial data selects from among a set of (potentially high-dimensional) covariates to associate with microbial taxon or pathway abundances. Mixed-effects linear models using a variance-stabilizing arcsin square root transform on relative abundances are then used to determine the significance of putative associations from among this reduced set. In the models, subject ID was used as a random effect, and the age of sample collection, mode of delivery, clinical centre (for cohort-wide comparisons), breastfeeding status (ongoing or stopped), solid food status (binary variable indicating whether solid food was introduced in the diet), number of sequencing reads and case–control outcome were used as fixed effects. Nominal *P* values were adjusted using the Benjamini–Hochberg FDR method. Here, microbial features with corrected *q* < 0.25 were reported. For metabolic pathways, pseudocount 2^6^ was added to CPM values to stabilize the variation in lowly abundant and/or prevalent but highly variable categories, and data were log_2_-transformed.

As previously described^40^, to associate microbial diversity with covariates while accounting for nonlinear, age-dependent effects, we first fitted a sigmoid function (nls function in R) to account for the longitudinal trend. Residuals of this model were then used as inputs for a mixed-effect model (glmmPQL function in the MASS R package), with subject IDs as random effects to account for repeated measurements in the data. Other factors were included in the model as fixed effects, and their significance levels were evaluated using *P* values reported by the model (Supplementary Table 2).

The association between T1D case–control outcome and microbial alpha diversity in individual clinical centres was tested using a linear mixed-effects model (glmmPQL function in MASS R package) on samples 730 days or less before T1D diagnosis. In the model, the age at stool sample collection and T1D case–control outcome were used as fixed effects, and subject ID was used as a random effect.

### Microbial variance explained by clinical and other covariates

Variance analysis was conducted using the adonis function in the vegan R package given a Bray–Curtis dissimilarity matrix of the taxonomic profiles and all TEDDY clinical metadata listed above. In brief, adonis conducts multivariate ANOVA using the dissimilarity matrix (that is, partitions the sums of squares) given the metadata as covariates. Statistical significance of the fit was assessed using permutation tests.

### HMO gene homology

The HMO gene cluster homologues between *B. longum* subsp. *infantis* and multiple taxa were analysed as follows. UniRef90 gene families corresponding to the protein sequences in the *B. longum* subsp. *infantis* HMO gene cluster^39^ (protein sequences Blon_2331-Blon_2361 in NCBI protein sequence database) were identified by translated BLAST search against ChocoPhlAn pangenome collection^48^ used by HUMAnN2. Identified hits were further filtered by requiring ≥50% alignment identity and ≥80% mutual coverage. Combining this information with HUMAnN2 species-stratified UniRef90 gene family quantification enabled calling these genes present given that they had sufficient read coverage, here defined as log_10_(counts per million) > 0.1 in at least 50 samples collected during breastfeeding. Differential gene prevalence during breastfeeding was tested using the samples in which the carrier species had >1% relative abundance. Testing was conducted using the test of equal or given proportions (prop.test function in R) and by comparing the prevalence (proportion of the samples for which the species in question harboured the gene according to the metagenomic data) of the gene in samples collected during breastfeeding with the samples collected after weaning. *P* values were adjusted for multiple testing by Benjamini–Hochberg method (p.adjust function in R). All homologues together with their BLAST search metrics, prevalence in the metagenomic data and corresponding *B. infantis* HMO gene are reported in Supplementary Table 5.

### Bacterial growth assays

*Bifidobacterium bifidum* strain RJX-1201, *Bifidobacterium breve* RJX-1202 and *Bifidobacterium longum* RJX-1203 were streaked on brain heart infusion agar (BD) supplemented with 1% vitamin K/hemin solution (BD; sBHI), and incubated for 48 h in a vinyl anaerobic chamber (Coy Laboratory Products) containing 5% CO_2_, 5% H_2_ and 90% N_2_ and maintained at 37 °C. Cells were transferred to sBHI liquid medium (BHI broth, BD, supplemented as above) and grown for 24 h in anaerobic conditions. Cultures were washed twice with PBS and optical density at 600 nm (OD_600_) was measured using a BioTek PowerWave 340 plate reader. OD_600_ was normalized to 0.2 for all strains and 5 μl bacteria inoculum was added to a final volume of 200 μl containing 10% sBHI and 125 mM carbon source (glucose, fucose, galactose or lactose) in a 96-well plate. OD_600_ was measured in the plate reader every hour for 48 h with 5 s of medium shaking before each measurement. All of the measurements were normalized to a medium-only blank. Experiment was repeated three times (*n* = 3) in triplicate and one representative experiment is shown. Error bars are s.d. of three technical replicates.

### Reporting summary

Further information on research design is available in the Nature Research Reporting Summary linked to this paper.

### Code availability

Code for Random Forest case–control comparisons and cohort wide MaAsLin association analyses in Supplementary Table 4 has been made publicly available at https://github.com/tvatanen/broad_teddy_microbiome_analyses. Other analysis software including quality control, taxonomic, and functional profilers is publicly available and referenced as appropriate.

## Online content

Any methods, additional references, Nature Research reporting summaries, source data, statements of data availability and associated accession codes are available at 10.1038/s41586-018-0620-2.

### Supplementary information

Supplementary Information This file contains Supplementary Notes 1-6, full legends for Supplementary Tables 1-5, Supplementary References and a list of members of the TEDDY Study Group.

Reporting Summary

Supplementary Table 1 Variance of taxonomic profiles explained by clinical covariates in cross-sectional adonis analysis – see Supplementary Information document for full description.

Supplementary Table 2 Associations between microbial alpha-diversity and clinical covariates – see Supplementary Information document for full description.

Supplementary Table 3 Variance of pathway profiles explained by clinical covariates in cross-sectional adonis analysis – see Supplementary Information document for full description.

Supplementary Table 4 Associations between the microbiome and case-control outcome in IA and T1D cohorts in TEDDY microbiome study – see Supplementary Information document for full description.

Supplementary Table 5 Homologs of the genes belonging to *B. infantis* HMO gene cluster across gut taxa in TEDDY metagenomic data – see Supplementary Information document for full description.

## Figures and Tables

**Extended Data Table 1:**
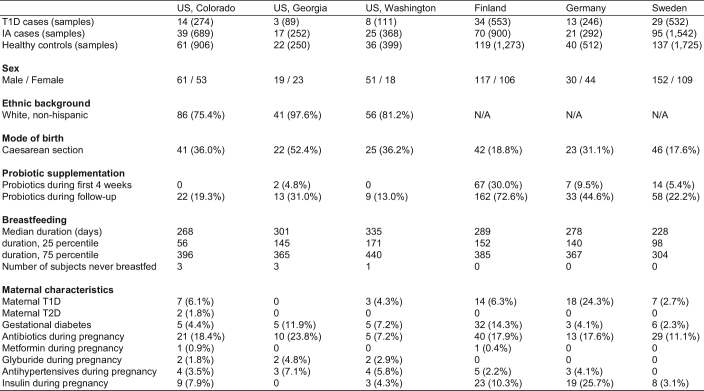
Summary of TEDDY microbiome cohort

Data on subjects’ ethnic background were not systematically collected in European clinical centres but these study populations were predominantly white, non-Hispanic. Reported antihypertensive drugs were atenolol (*n* = 2), bisoprolol (*n* = 1), labetalol (*n* = 6), methyldopa (*n* = 1), methyldopa plus methyldopate (*n* = 3), metoprolol (*n* = 4) and nifedipine (*n* = 5). No use of angiotensin-converting enzyme (ACE) inhibitors was reported. Numbers indicate the number of subjects (*n*) if not specified otherwise.

**Extended Data Table 2:**
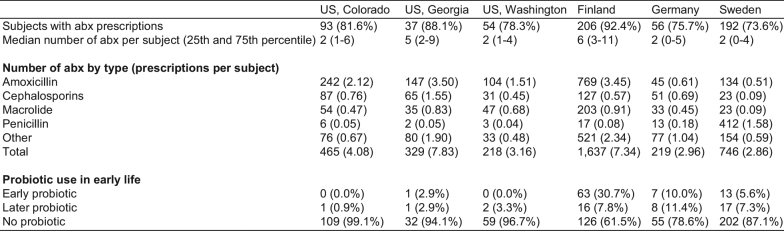
Antibiotics and probiotics

Top, 3,678 antibiotic prescriptions in the TEDDY microbiome study population by clinical centre. Bottom, early probiotic supplementation in TEDDY clinical centres. Probiotic use was stratified into three categories: probiotics during the first 4 weeks of life (early probiotic); probiotics before the first stool sample (roughly at three months) but not the first 4 weeks (later probiotic); and no probiotics before the first stool sample (no probiotic). Data for probiotics are presented as *n* (percentage).

## Data Availability

TEDDY microbiome 16S and whole-genome sequencing data that support the findings of this study are available in the NCBI database of Genotypes and Phenotypes (dbGaP) with the primary accession code phs001443.v1.p1, in accordance with the dbGaP controlled-access authorization process. Clinical metadata analysed during the current study are available in the NIDDK Central Repository at https://www.niddkrepository.org/studies/teddy.
