# Supplementary material for: The human gut microbiome in early-onset type 1 diabetes from the TEDDY study
Source: Nature. 2018 Oct 24;562(7728):589–94. doi: 10.1038/s41586-018-0620-2 (PMC6296767; doi:10.1038/s41586-018-0620-2)
Supplement: Supplementary file 1 — This file contains Supplementary Notes 1-6, full legends for Supplementary Tables 1-5, Supplementary References and a list of members of the TEDDY Study Group. [file 41586_2018_620_MOESM1_ESM.pdf]

nature
SUPPLEMENTARY INFORMATION
https://doi.org/10.1038/s41586-018-0620-2
In the format provided by the authors and unedited.

The human gut microbiome in early-onset type 1 diabetes from the TEDDY study

Tommi Vatanen[1]*, Eric A. Franzosa[1,2], Randall Schwager[2], Surya Tripathi[1], Timothy D. Arthur[1], Kendra Vehik[3], Åke Lernmark[4], William A. Hagopian[5], Marian J. Rewers[6], Jin-Xiong She[7], Jorma Toppari[8,9], Anette-G. Ziegler[10,11,12], Beena Akolkar[13], Jeffrey P. Krischer[3], Christopher J. Stewart[14,15], Nadim J. Ajami[14], Joseph F. Petrosino[14], Dirk Gevers[1,19], Harri Lähdesmäki[16], Hera Vlamakis[1], Curtis Huttenhower[1,2,20]* & Ramnik J. Xavier[1,17,18,20]*

[1]Broad Institute of MIT and Harvard, Cambridge, MA, USA. [2]Department of Biostatistics, Harvard T. H. Chan School of Public Health, Boston, MA, USA. [3]Health Informatics Institute, Morsani College of Medicine, University of South Florida, Tampa, FL, USA. [4]Department of Clinical Sciences, Lund University/CRC, Skåne University Hospital SUS, Malmo, Sweden. [5]Pacific Northwest Research Institute, Seattle, WA, USA. [6]Barbara Davis Center for Childhood Diabetes, University of Colorado, Aurora, CO, USA. [7]Center for Biotechnology and Genomic Medicine, Medical College of Georgia, Augusta University, Augusta, GA, USA. [8]Department of Pediatrics, Turku University Hospital, Turku, Finland. [9]Institute of Biomedicine, Research Centre for Integrative Physiology and Pharmacology, University of Turku, Turku, Finland. [10]Institute of Diabetes Research, Helmholtz Zentrum München, Munich, Germany. [11]Forschergruppe Diabetes, Technische Universität München, Klinikum Rechts der Isar, Munich, Germany. [12]Forschergruppe Diabetes e.V. at Helmholtz Zentrum München, Munich, Germany. [13]National Institute of Diabetes & Digestive & Kidney Diseases, Bethesda, MD, USA. [14]Alkek Center for Metagenomics and Microbiome Research, Department of Molecular Virology and Microbiology, Baylor College of Medicine, Houston, TX, USA. [15]Institute of Cellular Medicine, Newcastle University, Newcastle upon Tyne, UK. [16]Department of Computer Science, Aalto University, Espoo, Finland. [17]Gastrointestinal Unit, Center for the Study of Inflammatory Bowel Disease, and Center for Computational and Integrative Biology, Massachusetts General Hospital and Harvard Medical School, Boston, MA, USA. [18]Center for Microbiome Informatics and Therapeutics, MIT, Cambridge, MA, USA. [19]Present address: Janssen Human Microbiome Institute, Janssen Research and Development, Cambridge, MA, USA. [20]These authors jointly supervised this work: Curtis Huttenhower, Ramnik J. Xavier. *e-mail: vatanen@broadinstitute.org; chuttenh@hsph.harvard.edu; xavier@molbio.mgh.harvard.edu

© 2018 Springer Nature Limited. All rights reserved.

This PDF contains:

Supplementary Note 1: Microbial community dynamics in early life

Supplementary Note 2: Antibiotics interact with early microbial development

Supplementary Note 3: Functional dynamics of the early gut microbiome

Supplementary Note 4: IA and T1D cases are separated from controls by region-specific microbial features

Supplementary Note 5: Early probiotics are detectable in stool

Supplementary Note 6: Gut microbiomes' multifaceted role in IA and T1D

References for Supplementary Notes

Supplementary Table Legends

Members of the TEDDY Study Group

Other Supplementary Material includes:

Supplementary Tables 1-5

Supplementary Note 1: Microbial community dynamics in early life

To assess the dynamics of the developing gut microbiome through its detailed taxonomic composition, we analyzed the metagenomes using MetaPhlAn 2.0 and investigated the resulting species-level profiles by their beta diversities as measured by Bray-Curtis dissimilarity (**Fig. 2**). Principal coordinate analysis (PCoA) ordination of the beta diversities showed a strong longitudinal gradient and significant heterogeneity among the earliest samples. We identified four different types of early profiles, three of which were characterized by high abundance of one of three *Bifidobacterium* species (*B. bifidum*, *B. longum*, and *B. breve*) and corresponded to the clusters 1-3 determined by Dirichlet Multinomial Mixture modeling of the metagenomic data[1]. The fourth cluster was characterized by larger-than-typical abundance of phylum Proteobacteria. We noted some differences in how subjects occupied these clusters. For example, Finnish children tended to have more *B. breve* (Mixed effects logistic regression, p = 2.3e-5) and less *B. longum* (p = 0.0028) during the first year of life compared to children from other clinical centers (**Extended Data Fig. 1e,f**), and samples collected within a month after an antibiotic course tended to have higher levels of Proteobacteria (p = 7.1e-7).

To determine whether part of these observations could be explained by differential substrate availability, we tested the ability of the three *Bifidobacterium* species (*B. bifidum*, *B. longum*, and *B. breve*) isolated from infant stool samples to grow in low-nutrient medium containing various mono- or disaccharides common in the infant gut (glucose, galactose, lactose and fucose). When cultured with the addition of a single carbon source, each species displayed a unique profile of sugar utilization (**Extended Data Fig. 1g-k**). In glucose, while *B. breve* reached a maximal cell density before *B. longum*, eventually *B. longum* achieved a comparable final density (**Extended**

Data Fig. 1g). Between the three species, *B. breve* grew best on galactose (**Extended Data Fig. 1h**). We observed minimal growth for all species on fucose (**Extended Data Fig. 1i**). Both *B. breve* and *B. longum* grew on lactose, with *B. breve* growing faster initially, but *B. longum* reaching a higher final cell density (**Extended Data Fig. 1j**). While *B. bifidum* grew poorly in all of the sugars that we tested, it achieved a similar final density to the other species in rich medium (100% BHI, **Extended Data Fig. 1k**). These data, consistent with other studies analyzing carbohydrate preference from *Bifidobacterium* species[2-4], support the hypothesis that nutrient availability plays a key role in defining the dominant species in a microbial community.

We used PERMANOVA (as implemented in adonis function in vegan R package) to test for omnibus association between the taxonomic profiles and early life covariates. Inter-subject differences explained 35% of microbial taxonomic variation (permutation test, $p < 0.001$, 1,000 permutations), followed by age at stool sampling at roughly 4% of variance ($p < 0.001$). Since most other metadata are confounded by either subject ID or age, we next repeated the analysis cross-sectionally in three-month time intervals using only one stool sample per subject. This revealed that clinical center and breastfeeding were the only other factors affecting the gut microbial profiles with clear statistical significance (**Table S1**). Depending on the cross-section used, clinical center explained 1.6-3.1% of microbial variation (increasing over time), whereas 1.0-1.8% of the variation during the first year of life was explained by breastfeeding status (ongoing versus stopped). Other factors, namely mode of birth, introduction of meat or vegetables in diet, antibiotic treatments and technical variable sequencing depth showed effects with at most borderline nominal significance. These findings demonstrate that while multiple factors influence the developing microbiota in early childhood, only a very limited set of variables measured here -

namely inter-individual differences, geographic location, age, and breastfeeding - had strong and systematic effects distinguishable by linear adonis modeling.

To better understand the dynamics and individuality of developing gut microbiomes, we next interrogated the stability or self-similarity of the microbiomes longitudinally, both within subjects and across subjects (stratified by comparisons within and across clinical centers; **Fig. 2b**). When stability was measured by Bray-Curtis dissimilarity of species-level taxonomic profiles, the gap between individual stability and similarity within or across clinical centers was largest at the beginning of the sampling period, indicating that the children had particularly dissimilar microbiotas during these early months. Microbial similarity across individuals showed a similarly increasing trend when measured by Jaccard index (**Extended Data Fig. 2a**). While the difference in stability within and between clinical centers was small in comparison to the within-subject stability, the regional trends in the microbiome were still evident in terms of stability and in adonis modeling above (**Table S1**, **Fig. 2b, Extended Data Fig. 2a**). Early taxonomic manifestations of these regional differences included, for example, a tendency to have more *Ruminococcus gnavus* in Sweden and higher levels of *Lactobacillus rhamnosus* and *Veillonella parvula* in Finland (**Extended Data Fig. 2b-d**).

Microbial alpha-diversity increases during early childhood, with the majority of diversity accumulating during the second six months of life when the microbiome begins development towards an adult-like composition[5] (**Extended Data Fig. 3a**). To investigate more specifically which factors contribute to this early accrual of microbial diversity, we tested Shannon's diversity index of taxonomic profiles for associations with collected metadata (**Table S2**). Early breast milk

(repeated measures ANOVA, p = 4.6e-44) and formula (p = 7.5e-20) consumption were the most important factors contributing to the increase in diversity during early infancy. We also found differences in microbial diversity between the clinical centers (p = 3.2e-8, e.g. higher diversity in Sweden), between children born by vaginal birth versus caesarean section (p = 0.025), and between children who consumed probiotics versus those who did not (p = 8.0e-4). Similar to breastfeeding, probiotics were associated with slightly lower microbial diversity, an effect that could be caused by the introduction of a small set of bacteria that compete with the existing community early in life. This data did not show any associations between maternal diabetes (T1D, T2D or gestational diabetes) or maternal medications during pregnancy (antihypertensives, insulin) and gut microbial alpha diversity in offspring. In these data, the introduction of solid food had no significant association with microbial diversity; rather, the cessation of breastfeeding had the largest effect (ANOVA, partial $\eta^2 = 0.053$).

Previous longitudinal case-control studies in Finnish and Estonian children[6,7] have reported a drop in alpha-diversity in subjects who progressed to T1D in comparison to control subjects with no IA or subjects who seroconverted but had not yet progressed to T1D. Other comparable human cohort studies have not found such difference in microbial diversity[8,9]. In the present study, IA or T1D outcome was not associated with microbial diversity in either T1D or IA case-control cohorts (**Extended Data Fig. 3b,c**). We also interrogated clinical centers separately but did not see any difference between T1D cases and their controls prior to T1D onset in Finland (**Extended Data Fig. 3d**). Anecdotally, three T1D cases from the state of Georgia showed a reduced microbial alpha-diversity compared to their controls beginning two years prior to T1D diagnosis (**Extended**

Data Fig. 3e, N = 3 case-control pairs, 142 stool samples, mixed effects linear model, nominal p = 0.003).

Supplementary Note 2: Antibiotics interact with early microbial development

Oral antibiotics greatly perturb the gut microbiome, although details differ among individuals and in early life versus adulthood[10-12]. To investigate how antibiotics affected subjects in this cohort, we analyzed antibiotic prescriptions in TEDDY during the first three years of life (**Fig. 2c,d, Extended Data Table 2**). We measured microbial stability within subjects for pairs of consecutive samples (< 50 days apart) in two groups: those spanning an oral antibiotic exposure (n=654) and those with no antibiotics (n=6,734). By measuring microbial stability with respect to the age at sample collection, we detected a marked drop in stability in sample pairs when samples were separated by a course of antibiotics (**Fig. 2c**, **Extended Data Fig. 4a**). The effect of antibiotics on microbial stability was larger [effect size (difference of medians in **Extended Data Fig. 4a**) 0.22 in first 6 months and 0.067 at year two (months 24-29)] in the earliest comparisons; conversely, although antibiotics have been reported to induce loss of microbial diversity in adults, we found that they had a relatively modest impact on microbial diversity in early life (**Extended Data Fig. 4b,c**), similar to other infant cohorts[10,12]. Together, these findings suggest that any individual, not-yet-developed microbial community configuration in early life can be more dramatically disrupted prior to establishment of a stable, established community, but that the development process itself can rapidly re-continue after antibiotic perturbation. We also examined whether different types of antibiotics had different effects on microbial stability but did not observe significant differences (**Extended Data Fig. 4d,e**).

Supplementary Note 3: Functional dynamics of the early gut microbiome

The functional profiles were subjected to similar adonis and stability analyses as described above for taxonomic profiles. Twenty-four percent of variation in microbial pathway abundances in these data was explained by inter-subject differences (permutation test, $p < 0.001$, 1,000 permutations): a smaller amount than taxonomic variation, but still substantial, especially in very early life. The only covariates reaching statistical significance in the cross-sectional adonis analysis for the metabolic pathways were breastfeeding in an early cross-sections at 3 and 6 months explaining 4.4% and 1.3% of variance, respectively, and introduction of solid food explaining 1.1% of variance at 3 months (**Table S3**). Finally, when functional developmental dynamics were summarized using overall Bray-Curtis similarity within subject over time, individuals were again consistently more functionally similar than taxonomically, but with persistent differences that only gradually shifted to more adult-like configurations over the first several years of life (**Extended Data Fig. 5a**).

As discussed in the main text, inter-individual microbial community functional profiles tended to be more consistent than taxonomic profiles, due in part to the presence of "core" microbial community functions. These can include a range of processes broadly distributed across either the whole microbial tree of life, those enriched in host-associated microbes generally (including the developing infant gut), and niche-specific functions that may appear in a variety of different organisms but are adaptive for the gut community in particular (such as host immune system interaction). While broadly distributed functions are expected to remain stable over time, both niche-specific and completely non-core functions may fluctuate in metagenomic abundance in

response to environmental variation, such as changes in the gut environment during childhood development.

To characterize the subset of broadly distributed but dynamically changing microbial functions potentially important in the early gut development, we identified molecular functions that changed consistently within-subject prior to the age of one year. Focusing on subjects' enzyme abundance profiles, we selected each subject's earliest time point (month 3), their time point closest to year 1, and their time point closest to year 2 (when available). Across subjects with paired month-3:year-1 samples, we ranked enzymes according to the consistency of their change in abundance between these two time points. Specifically, each enzyme was scored according to the fraction of sample pairs where it increased minus the fraction where it decreased, restricted to sample pairs where the enzyme's community-wide abundance exceeded 10 copies per million (CPM) in at least one of the two samples. The abundances of these enzymes were then stratified according to contributing species to dissect relationships between taxonomic and functional changes.

The microbial enzymes that changed most consistently between month 3 and year 1 were clearly influenced by underlying taxonomic changes, with enzymes enriched among the Bifidobacteria highly abundant at month 3 and less-so thereafter (**Fig. 3**). Among the most consistently declining enzymes was L-lactate dehydrogenase (1.1.1.27), which has been well characterized in Bifidobacteria for its role in the fermentation of milk[13]. This change is notably coincident with changes in breastfeeding status, with 73% of subjects breastfed at the time of their month 3 sample and 28% at year 1. Ribokinase (2.7.1.15) also exhibited a marked decline in abundance over this time period, and has been proposed as a mechanism for harvesting ribose from the human gut as a

carbon source in *Bifidobacterium breve*[14]. The fact that these enzymes are largely undetected in later-colonizing species suggests that they are specifically adaptive to the metabolic environment of the early human gut environment and may facilitate early colonization by *Bifidobacterium* species.

Conversely, a second set of enzymes (largely depleted at month 3) becomes more abundant in years 1 and 2 (**Fig. 3**). These enzymes were, broadly, elements of anaerobic metabolism most dominantly contributed by *Ruminococcus gnavus*, *R. bromii*, and *Faecalibacterium prausnitzii*, which herald the transition to a more adult-like gut microbiome. Notably, coincident with the decline in breastfeeding between month 3 and year 1, infants transitioned from 53% having tried solid food to 100%. Thus, expansion of enzymatic functions may reflect a microbial community reaction to more variable, fermentative energy sources. For example, transketolase (2.2.1.1) consistently increased in abundance between month 3 and year 1 and has been implicated in the metabolism of fiber (via pentose sugars) by ruminococci[15]. The glycolytic enzyme 6-phosphofructokinase (2.7.1.11) also increased over this interval. Thus, in a background of broadly conserved functions, prevalent gut-specific functions, and individualized differences, a number of potentially host-diet-associated microbial functions are changing clearly and consistently within-subject between month 3 and year 1.

Supplementary Note 4: IA and T1D cases are separated from controls by region-specific microbial features

Random Forest (RFs) classifying between case and control samples were trained using samples prior to T1D diagnoses and using 2,000 trees per forest using the randomForest package in R.

Training data included full taxonomic or functional profiles together with age of sample collection and clinical center. Overfitting of the model, caused by the repeated measures data (samples are not i.i.d.), was compensated by using leave-one-out cross-validation, where one case-control pair was held out as separate validation data at a time. Bootstrapping in RF training was stratified by case-control class in such a way that each bootstrap sample had same number of case and control samples (**Extended Data Fig. 5a**).

In 100 T1D case-control pairs, RFs achieved a median error rate across cross-validation folds of 0.449 for taxonomic profiles and 0.450 for functional profiles (**Extended Data Fig. 6a**), when leaving out one case-control pair at a time for cross-validation. This suggests, at most, a weak microbiome signal of either type separating T1D cases from controls. Furthermore, all case-control comparisons were impeded by site-specific differences in the microbiome. For example, IA cases in Finland harbored more *Veillonella parvula* compared to their Finnish controls (mixed effects linear model, q=0.064), whereas Swedish IA cases had less *V. parvula* (q=0.055) and other unclassified *Veillonella* sequences (q=0.24) compared to their Swedish controls, highlighting the regional differences in the cohorts.

The TEDDY cohort includes progressors to clinical T1D ranging from the first year of life through age six. Other differences in the disease onset included prior appearance of a single or multiple persistent AABs, although the majority of the single AAB positive subjects (128 / 143) had not yet developed T1D. To explore whether these differences were linked to the gut microbiome, we first tested whether the microbiome in subjects diagnosed with T1D before the age of two differed from those of children who developed T1D later. However, this comparison did not show any

consistent differences between these groups of early and late progressors. We also compared subjects with a single or multiple AABs and healthy controls. The results of these analyses were often concordant between subjects with a single or multiple AABs, and with the other case-control comparisons above, but also revealed some additional differences in subjects with only a single persistent AAB. For example, Finnish subjects with one AAB had more *Ruminococcus gnavus* (**Extended Data Fig. 6b**, q=0.011) compared to both subjects with multiple AABs and healthy controls.

Compared to population level observations, most of the detected changes in cases versus controls were modest in effect size and statistical significance. Importantly, however, a part of them are concordant with the existing literature. The drop in microbial alpha diversity preceding T1D diagnosis in Georgia agrees with what has been reported in previous studies[6,7]. *B. vulgatus*, more abundant in IA cases in Sweden, is phylogenetically close relative to *B. dorei* which has been previously associated with T1D onset[16,17]. Most importantly, SCFAs have been linked to multiple facets of gut health in many recent studies[18]. They, for example, enhance the gut barrier[19] and participate in host-signaling mechanisms[20]. Diet modulates microbial SCFA production, by providing fibers for fermentation as well as by modulating the microbial community composition itself. Thus, any protective or predisposing dietary effects may, at least partly, be mediated through their contribution to microbial SCFA release.

Supplementary Note 5: Early probiotics are detectable in stool

Probiotics are of particular interest in early life due to their proposed ability to influence immune development during microbiome acquisition[21,22]. In this cohort, early probiotic supplementation

prior to the first collected stool sample (approximately age three months) was relatively common in Finland (38.5% of subjects received probiotics), less common in Germany (21.4%) and Sweden (12.9%), and unusual in the US (2.5%, **Extended Data Table 2**). We found that probiotics given during first four weeks of life or later, but before the first stool sample was collected at approximately three months, were associated with increased abundance and prevalence of two *Lactobacillus* species in the first stool sample, *L. reuteri* and *L. rhamnosus* (**Extended Data Fig. 6d,e**). These species are common in probiotic supplements. *L. rhamnosus* was detected in the first stool sample in 58.8% of infants who were given probiotics during first four weeks of life, whereas the percentage was only 29.3% in infants with no reported probiotics prior to the first stool sample. *L. reuteri* was detected in only 1.2% of infants with no reported probiotics but was more common (Fisher's exact test, p = 1.1e-12) when preceding probiotic supplementation was given.

Supplementary Note 6: Gut microbiomes' multifaceted role in IA and T1D

As discussed in the main text, most of the taxonomic and functional signals we detected in case-control comparisons were modest in effect size and statistical significance. This could be due to multiple reasons that should be considered in future investigations. First, T1D is a complex disease with heterogeneous etiology and multiple "subtypes" distinguished by differences in disease onset, implicating that healthy controls in this nested case-control cohort may also later progress to IA, T1D or both. For example, the age of disease onset has been decreasing for unclear reasons. Additionally, existing literature has identified both protective[23] and predisposing[24] microbial factors. Second, any signal, either predisposing or protective, may be temporally diffuse, making it statistically difficult to establish. Indeed, cross-sectional analyses did not show any separation between cases and controls, even when tested in one clinical center at a time. Third, the most

prominent trends in this data, interindividual and geographic differences, could in principle be due either to biological differences or technical variation. For example, there may have been systematic differences in sample collection between participating families and/or clinical centers, but our data does not allow distinguishing between these systematic biases and other variation. Fourth, signals in the microbiome may consist of some yet unknown or poorly characterized microbial taxa and functions, which our reference database-based analyses would neglect. Finally, the stool sample collection started around three months of age, leaving this study agnostic in terms of the initial microbial acquisition and community assembly during the first three months of life. These factors, together with the complex disease etiology, suggest that the microbiome has complex and multifaceted role in T1D that may be limited in overall impact.

In contrast to microbial covariates, genetic risk loci of substantial effect for T1D have been well-studied and are in large part contained within the human leukocyte antigen (HLA) class II genes HLA-DR and HLA-DQ[25]. In addition to T1D, variation in the HLA locus is an important predisposing factor in most immune-mediated and autoimmune diseases, and a protective element in some infectious diseases[26]; for example, inflammatory bowel disease (IBD) has strong associations with both HLA-DR and HLA-DQ loci. Evidence for other microbial associations with HLA loci, such as those with infectious disease (e.g. viral infections), is limited due to relatively small patient cohort sizes, but the overlap in genetic susceptibility loci suggests that infectious episodes and viruses can trigger autoimmunity[26]. Indeed, several studies posit that viral infections can trigger T1D while the strongest evidence supports the role of enteroviral infections in both animal models and humans[27]. Speculatively, the gut microbiome may play a role in this puzzle, for example, by maintaining the healthy gut barrier function through SCFA production thus barring

any infection-related toxins from entering the host circulation[28]. However, the role of the gut microbiome and possible mechanistic interactions with other genetic and environmental risk factors of human T1D remains to be proven.

Other non-HLA risk loci are shared between T1D and other immune diseases, particularly IBD, such as FUT2, CTLA-4 and PTPN2[29-31], even though the directionality of the associations are not always concordant among diseases[25]. FUT2, one of these shared genetic risk loci, determines the mucosal expression of blood group A and B antigen, i.e. secretor status, and non-secretors are susceptible to both T1D[32] and Crohn's disease (a subtype of IBD)[33,34]. Secretor status is also in turn associated with the gut microbial composition in both infants[35] and adults[36].

Finally, shared loci including FUT2 are of interest for their potential effects on specific early-life microbes, such as the role of secretor status in the acquisition of *Bifidobacterium* spp. Bifidobacterial colonization, selection and preservation are modulated by multiple factors such as maternal FUT2 genotype[35], molecular repertoire of breast milk, maternal gut and breast milk microbiome (via vertical transmission)[37], siblings, house pets and built environment. The clade has many important functional roles in the early gut microbiome[38]: protection against infectious diseases[39], improved gut barrier function[40], and possibly protection against allergic sensitization and inflammation[41]. Concordant with existing evidence[42,43], our data confirmed that certain *B. longum* strains specialized in HMO utilization are positively selected during breastfeeding but are reduced or disappear after weaning. Importantly, we also showed that many *Bifidobacterium* species are systematically reduced after oral antibiotics, which may have adverse effects on the host immune development.

References

1 Stewart, C. J. *et al.* Temporal development of the gut microbiome in early childhood from the TEDDY study. *Nature*, doi:10.1038/s41586-018-0617-x (2018).

2 Schwab, C. *et al.* Trophic Interactions of Infant Bifidobacteria and Eubacterium hallii during L-Fucose and Fucosyllactose Degradation. *Front Microbiol* **8**, 95, doi:10.3389/fmicb.2017.00095 (2017).

3 Palframan, R. J., Gibson, G. R. & Rastall, R. A. Carbohydrate preferences of Bifidobacterium species isolated from the human gut. *Curr Issues Intest Microbiol* **4**, 71-75 (2003).

4 Parche, S. *et al.* Lactose-over-glucose preference in Bifidobacterium longum NCC2705: glcP, encoding a glucose transporter, is subject to lactose repression. *J Bacteriol* **188**, 1260-1265, doi:10.1128/JB.188.4.1260-1265.2006 (2006).

5 Yatsunenko, T. *et al.* Human gut microbiome viewed across age and geography. *Nature* **486**, 222-227, doi:10.1038/nature11053 (2012).

6 Kostic, A. D. *et al.* The dynamics of the human infant gut microbiome in development and in progression toward type 1 diabetes. *Cell Host Microbe* **17**, 260-273, doi:10.1016/j.chom.2015.01.001 (2015).

7 Giongo, A. *et al.* Toward defining the autoimmune microbiome for type 1 diabetes. *ISME J* **5**, 82-91, doi:10.1038/ismej.2010.92 (2011).

8 Endesfelder, D. *et al.* Compromised gut microbiota networks in children with anti-islet cell autoimmunity. *Diabetes* **63**, 2006-2014, doi:10.2337/db13-1676 (2014).

9 Maffeis, C. *et al.* Association between intestinal permeability and faecal microbiota composition in Italian children with beta cell autoimmunity at risk for type 1 diabetes. *Diabetes Metab Res Rev* **32**, 700-709, doi:10.1002/dmrr.2790 (2016).

10 Yassour, M. *et al.* Natural history of the infant gut microbiome and impact of antibiotic treatment on bacterial strain diversity and stability. *Sci Transl Med* **8**, 343ra381, doi:10.1126/scitranslmed.aad0917 (2016).

11 Raymond, F. *et al.* The initial state of the human gut microbiome determines its reshaping by antibiotics. *ISME J* **10**, 707-720, doi:10.1038/ismej.2015.148 (2016).

12 Bokulich, N. A. *et al.* Antibiotics, birth mode, and diet shape microbiome maturation during early life. *Sci Transl Med* **8**, 343ra382, doi:10.1126/scitranslmed.aad7121 (2016).

13 O'Callaghan, A. & van Sinderen, D. Bifidobacteria and Their Role as Members of the Human Gut Microbiota. *Front Microbiol* **7**, 925, doi:10.3389/fmicb.2016.00925 (2016).

14 Pokusaeva, K. *et al.* Ribose utilization by the human commensal Bifidobacterium breve UCC2003. *Microb Biotechnol* **3**, 311-323, doi:10.1111/j.1751-7915.2009.00152.x (2010).

15 Thurston, B., Dawson, K. A. & Strobel, H. J. Pentose utilization by the ruminal bacterium Ruminococcus albus. *Appl Environ Microbiol* **60**, 1087-1092 (1994).

16 Davis-Richardson, A. G. *et al.* Bacteroides dorei dominates gut microbiome prior to autoimmunity in Finnish children at high risk for type 1 diabetes. *Front Microbiol* **5**, 678, doi:10.3389/fmicb.2014.00678 (2014).

17 Vatanen, T. *et al.* Variation in Microbiome LPS Immunogenicity Contributes to Autoimmunity in Humans. *Cell* **165**, 842-853, doi:10.1016/j.cell.2016.04.007 (2016).

18 Rios-Covian, D. *et al.* Intestinal Short Chain Fatty Acids and their Link with Diet and Human Health. *Front Microbiol* **7**, 185, doi:10.3389/fmicb.2016.00185 (2016).

19 Peng, L., Li, Z. R., Green, R. S., Holzman, I. R. & Lin, J. Butyrate enhances the intestinal barrier by facilitating tight junction assembly via activation of AMP-activated protein kinase in Caco-2 cell monolayers. *J Nutr* **139**, 1619-1625, doi:10.3945/jn.109.104638 (2009).

20 Smith, P. M. *et al.* The microbial metabolites, short-chain fatty acids, regulate colonic Treg cell homeostasis. *Science* **341**, 569-573, doi:10.1126/science.1241165 (2013).

21 Uusitalo, U. *et al.* Association of Early Exposure of Probiotics and Islet Autoimmunity in the TEDDY Study. *JAMA Pediatr* **170**, 20-28, doi:10.1001/jamapediatrics.2015.2757 (2016).

22 Cervantes-Barragan, L. *et al.* Lactobacillus reuteri induces gut intraepithelial CD4+CD8alphaalpha+ T cells. *Science* **357**, 806-810, doi:10.1126/science.aah5825 (2017).

23 Marino, E. *et al.* Gut microbial metabolites limit the frequency of autoimmune T cells and protect against type 1 diabetes. *Nat Immunol* **18**, 552-562, doi:10.1038/ni.3713 (2017).

24 Costa, F. R. *et al.* Gut microbiota translocation to the pancreatic lymph nodes triggers NOD2 activation and contributes to T1D onset. *J Exp Med* **213**, 1223-1239, doi:10.1084/jem.20150744 (2016).

25 Pociot, F. & Lernmark, A. Genetic risk factors for type 1 diabetes. *Lancet* **387**, 2331-2339, doi:10.1016/S0140-6736(16)30582-7 (2016).

26 Matzaraki, V., Kumar, V., Wijmenga, C. & Zhernakova, A. The MHC locus and genetic susceptibility to autoimmune and infectious diseases. *Genome Biol* **18**, 76, doi:10.1186/s13059-017-1207-1 (2017).

27 Rewers, M. & Ludvigsson, J. Environmental risk factors for type 1 diabetes. *Lancet* **387**, 2340-2348, doi:10.1016/S0140-6736(16)30507-4 (2016).

28 Kelly, C. J. *et al.* Crosstalk between Microbiota-Derived Short-Chain Fatty Acids and Intestinal Epithelial HIF Augments Tissue Barrier Function. *Cell Host Microbe* **17**, 662-671, doi:10.1016/j.chom.2015.03.005 (2015).

29 Barrett, J. C. *et al.* Genome-wide association study and meta-analysis find that over 40 loci affect risk of type 1 diabetes. *Nat Genet* **41**, 703-707, doi:10.1038/ng.381 (2009).

30 Concannon, P., Rich, S. S. & Nepom, G. T. Genetics of type 1A diabetes. *N Engl J Med* **360**, 1646-1654, doi:10.1056/NEJMra0808284 (2009).

31 Liu, J. Z. *et al.* Association analyses identify 38 susceptibility loci for inflammatory bowel disease and highlight shared genetic risk across populations. *Nat Genet* **47**, 979-986, doi:10.1038/ng.3359 (2015).

32 Smyth, D. J. *et al.* FUT2 nonsecretor status links type 1 diabetes susceptibility and resistance to infection. *Diabetes* **60**, 3081-3084, doi:10.2337/db11-0638 (2011).

33 McGovern, D. P. *et al.* Fucosyltransferase 2 (FUT2) non-secretor status is associated with Crohn's disease. *Hum Mol Genet* **19**, 3468-3476, doi:10.1093/hmg/ddq248 (2010).

34 Wu, H. *et al.* Association of Fucosyltransferase 2 Gene Polymorphisms with Inflammatory Bowel Disease in Patients from Southeast China. *Gastroenterol Res Pract* **2017**, 4148651, doi:10.1155/2017/4148651 (2017).

35 Lewis, Z. T. *et al.* Maternal fucosyltransferase 2 status affects the gut bifidobacterial communities of breastfed infants. *Microbiome* **3**, 13, doi:10.1186/s40168-015-0071-z (2015).

36 Wacklin, P. *et al.* Secretor genotype (FUT2 gene) is strongly associated with the composition of Bifidobacteria in the human intestine. *PLoS One* **6**, e20113, doi:10.1371/journal.pone.0020113 (2011).

37 Asnicar, F. *et al.* Studying Vertical Microbiome Transmission from Mothers to Infants by Strain-Level Metagenomic Profiling. *mSystems* **2**, doi:10.1128/mSystems.00164-16 (2017).

38 Lewis, Z. T. & Mills, D. A. Differential Establishment of Bifidobacteria in the Breastfed Infant Gut. *Nestle Nutr Inst Workshop Ser* **88**, 149-159, doi:10.1159/000455399 (2017).

39 Fukuda, S. *et al.* Bifidobacteria can protect from enteropathogenic infection through production of acetate. *Nature* **469**, 543-547, doi:10.1038/nature09646 (2011).

40 Chichlowski, M., De Lartigue, G., German, J. B., Raybould, H. E. & Mills, D. A. Bifidobacteria isolated from infants and cultured on human milk oligosaccharides affect intestinal epithelial function. *J Pediatr Gastroenterol Nutr* **55**, 321-327, doi:10.1097/MPG.0b013e31824fb899 (2012).

41 Kirjavainen, P. V., Arvola, T., Salminen, S. J. & Isolauri, E. Aberrant composition of gut microbiota of allergic infants: a target of bifidobacterial therapy at weaning? *Gut* **51**, 51-55 (2002).

42 Sela, D. A. *et al.* The genome sequence of Bifidobacterium longum subsp. infantis reveals adaptations for milk utilization within the infant microbiome. *Proc Natl Acad Sci U S A* **105**, 18964-18969, doi:10.1073/pnas.0809584105 (2008).

43 Mattarelli, P., Bonaparte, C., Pot, B. & Biavati, B. Proposal to reclassify the three biotypes of Bifidobacterium longum as three subspecies: Bifidobacterium longum subsp. longum subsp. nov., Bifidobacterium longum subsp. infantis comb. nov. and Bifidobacterium longum subsp. suis comb. nov. *Int J Syst Evol Microbiol* **58**, 767-772, doi:10.1099/ijs.0.65319-0 (2008).

Supplementary Table Legends

Supplementary Table 1. Variance of taxonomic profiles explained by clinical covariates in cross-sectional adonis analysis. Bray-Curtis dissimilarity matrix of the taxonomic profiles was modeled using adonis analysis to assess the proportion of variance explained by different covariates. Table shows R-squared (the proportion of variance explained), nominal p-value based on permutation test (100,000 permutations, two-sided), and FDR corrected p-value (q-value) for covariates in each cross-section used.

Supplementary Table 2. Associations between microbial alpha-diversity and clinical covariates. Associations between microbial alpha diversity (N = 10,250 samples) and clinical covariates were evaluated using a linear mixed effects model with subject ID as random effect and other clinical covariates as fixed effects using glmmPQL function in MASS package in R. The columns of the table are explained in the Excel file.

Supplementary Table 3. Variance of pathway profiles explained by clinical covariates in cross-sectional adonis analysis. Bray-Curtis dissimilarity matrix of the pathway profiles was modeled using adonis analysis to assess the proportion of variance explained by different covariates. Table shows R-squared (the proportion of variance explained), nominal p-value based on permutation test (100,000 permutations, two-sided), and FDR corrected p-value (q-value) for covariates in each cross-section used.

Supplementary Table 4. Associations between the microbiome and case-control outcome in IA and T1D cohorts in TEDDY microbiome study. Association testing was conducted using

linear mixed effect models in MaAsLin. Nominal p-values were adjusted using the Benjamini-Hochberg false discovery rate (FDR) method. The columns of the table are explained in the Excel file.

Supplementary Table 5. Homologs of the genes belonging to *B. infantis* HMO gene cluster across gut taxa in TEDDY metagenomic data. UniRef90 gene families corresponding to the HMO genes were identified by translated BLAST search against ChocoPhlAn pangenome collection and filtered by requiring >=50% identity and >=80% mutual coverage. Sheet "HMO homolog frequencies" contains the frequencies of all homologs to *B. infantis* HMO genes per bacterial species. Sheet "alignment details" reports the hits (Gene family) together with the carrier species (Species) and corresponding B. infantis gene (B. infantis HMO gene) occurring in more than 50 samples in TEDDY metagenomic data. The table also shows the prevalence of these genes in TEDDY metagenomic data (# of samples with gene family present) as well as how many of these samples were collected during breastfeeding period (# of samples during breastfeeding). The difference in prevalence of each gene during breastfeeding (Gene family prevalence during breastfeeding) and after weaning (Gene family prevalence after weaning) was tested using the test of proportions (two-sided, prop.test function in R), and the corresponding p-values and Benjamini-Hochberg FDR corrected q-values are reported.

Members of the TEDDY Study Group

Colorado Clinical Center: Marian Rewers, M.D., Ph.D., PI[1,4,5,6,10,11], Kimberly Bautista[12], Judith Baxter[9,10,12,15], Ruth Bedoy[2], Daniel Felipe-Morales, Kimberly Driscoll, Ph.D.[9], Brigitte I. Frohnert, M.D.[2,14], Marisa Gallant, M.D.[13], Patricia Gesualdo[2,6,12,14,15], Michelle Hoffman[12,13,14], Rachel Karban[12], Edwin Liu, M.D.[13], Jill Norris, Ph.D.[2,3,12], Adela Samper-Imaz, Andrea Steck, M.D.[3,14], Kathleen Waugh[6,7,12,15], Hali Wright[12]. University of Colorado, Anschutz Medical Campus, Barbara Davis Center for Childhood Diabetes.

Finland Clinical Center: Jorma Toppari, M.D., Ph.D., PI[¥^1,4,11,14], Olli G. Simell, M.D., Ph.D., Annika Adamsson, Ph.D.[^12], Suvi Ahonen*[±§], Heikki Hyöty, M.D., Ph.D.*[±6], Jorma Ilonen, M.D., Ph.D.[¥¶3], Sanna Jokipuu[^], Tiina Kallio[^], Leena Karlsson[^], Miia Kähönen[µ¤], Mikael Knip, M.D., Ph.D.*[±5], Lea Kovanen*[±§], Mirva Koreasalo*[±§2], Kalle Kurppa, M.D., Ph.D.*[±13], Tiina Latva-aho[µ¤], Maria Lönnrot, M.D., Ph.D.*[±6], Elina Mäntymäki[^], Katja Multasuo[µ¤], Tiina Niininen[±]*[12], Sari Niinistö[±§2], Mia Nyblom*[±], Petra Rajala[^], Jenna Rautanen[±§], Anne Riikonen*[±§], Minna Romo[^], Juulia Rönkä[µ¤], Satu Simell, M.D., Ph.D.[¥13], Tuula Simell, Ph.D.[¥12], Maija Sjöberg[¥^12,14], Aino Stenius[µ¤12], Maria Leppänen[^], Sini Vainionpää[^], Eeva Varjonen[¥^12], Riitta Veijola, M.D., Ph.D.[µ¤14], Suvi M. Virtanen, M.D., Ph.D.*[±§2], Mari Vähä-Mäkilä[^], Mari Åkerlund*[±§], Katri Lindfors, Ph.D.*[13] [¥]University of Turku, *University of Tampere, [µ]University of Oulu, [^]Turku University Hospital, Hospital District of Southwest Finland, [±]Tampere University Hospital, [¤]Oulu University Hospital, §National Institute for Health and Welfare, Finland, [¶]University of Kuopio.

Georgia/Florida Clinical Center: Jin-Xiong She, Ph.D., PI[1,3,4,11], Desmond Schatz, M.D.*[4,5,7,8], Diane Hopkins[12], Leigh Steed[12,13,14,15], Jennifer Bryant, Jamie Thomas*[6,12], Janey Adams*[12],

Katherine Silvis[2], Michael Haller, M.D.*[14], Melissa Gardiner, Richard McIndoe, Ph.D., Ashok Sharma, Stephen W. Anderson, M.D.[^], Laura Jacobsen, M.D.[*14] Center for Biotechnology and Genomic Medicine, Augusta University. *University of Florida, [^]Pediatric Endocrine Associates, Atlanta.

Germany Clinical Center: Anette G. Ziegler, M.D., PI[1,3,4,11], Andreas Beyerlein, Ph.D.[2], Ezio Bonifacio Ph.D.*[5], Anja Heublein, Michael Hummel, M.D.[13], Sandra Hummel, Ph.D.[2], Annette Knopff[7], Charlotte Koch, Sibylle Koletzko, M.D.[¶13], Claudia Ramminger, Roswith Roth, Ph.D.[9], Marlon Scholz, Laura Schulzik[2], Joanna Stock[9,12,14], Katharina Warncke, M.D.[14], Lorena Wendel, Christiane Winkler, Ph.D.[2,12,15]. Forschergruppe Diabetes e.V. and Institute of Diabetes Research, Helmholtz Zentrum München, Forschergruppe Diabetes, and Klinikum rechts der Isar, Technische Universität München. *Center for Regenerative Therapies, TU Dresden, [¶]Dr. von Hauner Children's Hospital, Department of Gastroenterology, Ludwig Maximillians University Munich.

Sweden Clinical Center: Åke Lernmark, Ph.D., PI[1,3,4,5,6,8,10,11,15], Daniel Agardh, M.D., Ph.D.[13], Carin Andrén Aronsson, Ph.D.[2,12,13], Maria Ask, Jenny Bremer, Ulla-Marie Carlsson, Corrado Cilio, Ph.D., M.D.[5], Emelie Ericson-Hallström, Annika Fors, Lina Fransson, Thomas Gard, Rasmus Bennet, Carina Hansson, Susanne Hyberg, Hanna Jisser, Fredrik Johansen, Berglind Jonsdottir, M.D., Silvija Jovic, Helena Elding Larsson, M.D., Ph.D. [6,14], Marielle Lindström, Markus Lundgren, M.D.[14], Maria Månsson-Martinez, Maria Markan, Jessica Melin[12], Zeliha Mestan, Caroline Nilsson, Karin Ottosson, Kobra Rahmati, Anita Ramelius, Falastin Salami, Sara Sibthorpe, Anette Sjöberg, Birgitta Sjöberg, Evelyn Tekum Amboh, Carina Törn, Ph.D. [3,15], Anne Wallin, Åsa Wimar[14], Sofie Åberg. Lund University

Washington Clinical Center: William A. Hagopian, M.D., Ph.D., PI[1,3,4, 5, 6,7,11,13, 14], Michael Killian[6,7,12,13], Claire Cowen Crouch[12,14,15], Jennifer Skidmore[2], Ashley Akramoff, Jana Banjanin, Masumeh Chavoshi, Kayleen Dunson, Rachel Hervey, Shana Levenson, Rachel Lyons, Arlene Meyer, Denise Mulenga, Davey Schmitt, Julie Schwabe. Pacific Northwest Research Institute.

Pennsylvania Satellite Center: Dorothy Becker, M.D., Margaret Franciscus, MaryEllen Dalmagro-Elias Smith[2], Ashi Daftary, M.D., Mary Beth Klein, Chrystal Yates. Children's Hospital of Pittsburgh of UPMC.

Data Coordinating Center: Jeffrey P. Krischer, Ph.D.,PI[1,4,5,10,11], Sarah Austin-Gonzalez, Maryouri Avendano, Sandra Baethke, Rasheedah Brown[12,15], Brant Burkhardt, Ph.D.[5,6], Martha Butterworth[2], Joanna Clasen, David Cuthbertson, Christopher Eberhard, Steven Fiske[9], Dena Garcia, Jennifer Garmeson, Veena Gowda, Kathleen Heyman, Belinda Hsiao, Francisco Perez Laras, Hye-Seung Lee, Ph.D.[1,2,13,15], Shu Liu, Xiang Liu, Ph.D.[2,3,9,14], Kristian Lynch, Ph.D. [5,6,9,15], Colleen Maguire, Jamie Malloy, Cristina McCarthy[12,15], Aubrie Merrell, Steven Meulemans, Hemang Parikh, Ph.D.[3], Ryan Quigley, Cassandra Remedios, Chris Shaffer, Laura Smith, Ph.D.[9,12], Susan Smith[12,15], Noah Sulman, Ph.D., Roy Tamura, Ph.D.[1,2,13], Ulla Uusitalo, Ph.D.[2,15], Kendra Vehik, Ph.D.[4,5,6,14,15], Ponni Vijayakandipan, Keith Wood, Jimin Yang, Ph.D., R.D.[2,15]. *Past staff: Michael Abbondondolo, Lori Ballard, David Hadley, Ph.D., Wendy McLeod.* University of South Florida.

Project scientist: Beena Akolkar, Ph.D.[1,3,4,5,6,7,10,11]. National Institutes of Diabetes and Digestive and Kidney Diseases.

Autoantibody Reference Laboratories: Liping Yu, M.D.[^5], Dongmei Miao, M.D.[^], Polly Bingley, M.D., FRCP*[5], Alistair Williams*, Kyla Chandler*, Claire Williams*, Gifty George*, Sian Grace*, Ben Gillard*. [^]Barbara Davis Center for Childhood Diabetes, University of Colorado Denver, *Bristol Medical School, University of Bristol UK.

HLA Reference Laboratory: William Hagopian[3], MD, PhD, Masumeh Chavoshi, Pacific Northwest Research Institute, Seatte WA. (Previously Henry Erlich, Ph.D.[3], Steven J. Mack, Ph.D., Anna Lisa Fear. Center for Genetics, Children's Hospital Oakland Research Institute.)

Metagenomics and Microbiome Laboratory: Joseph F. Petrosino, Ph.D.[6*], Nadim J. Ajami, Ph.D.[*], Richard E. Lloyd, Ph.D.[6*], Matthew C. Ross, Ph.D.[*], Jacqueline L. O'Brien, Ph.D.[*], Diane S. Hutchinson, Ph.D.[*], Daniel P. Smith, Ph.D.[*], Matthew C. Wong[*], Xianjun Tian, Ph.D.[*], Tulin Ayvaz[*], Auriole Tamegnon[*], Nguyen Truong[*], Hannah Moreno[*], Lauren Riley[*], Eduardo Moreno[*], Tonya Bauch[*], Lenka Kusic[*], Ginger Metcalf[^], Donna Muzny[^], HarshaVArdhan Doddapaneni, Ph.D.[^], Richard Gibbs, Ph.D.[^]. *Alkek Center for Metagenomics and Microbiome Research, Department of Molecular Virology and Microbiology, Baylor College of Medicine, ^Human Genome Sequencing Center, Baylor College of Medicine.

Repository: Sandra Ke, Niveen Mulholland, Ph.D. NIDDK Biosample Repository at Fisher BioServices.

Other contributors: Kasia Bourcier, Ph.D.[5], National Institutes of Allergy and Infectious Diseases. Thomas Briese, Ph.D.[6,15], Columbia University. Suzanne Bennett Johnson, Ph.D.[9,12], Florida State University. Eric Triplett, Ph.D.[6], University of Florida.

Committees:

[1]Ancillary Studies, [2]Diet, [3]Genetics, [4]Human Subjects/Publicity/Publications, [5]Immune Markers, [6]Infectious Agents, [7]Laboratory Implementation, [8]Maternal Studies, [9]Psychosocial, [10]Quality Assurance, [11]Steering, [12]Study Coordinators, [13]Celiac Disease, [14]Clinical Implementation, [15]Quality Assurance Subcommittee on Data Quality.
