# Supplementary material for: The human gut microbiome in early-onset type 1 diabetes from the TEDDY study
Source: Nature. 2018 Oct 24;562(7728):589–94. doi: 10.1038/s41586-018-0620-2 (PMC6296767; doi:10.1038/s41586-018-0620-2)
Supplement: Supplementary file 2 — Reporting Summary [file 41586_2018_620_MOESM2_ESM.pdf]

nature research

Corresponding author(s): Tommi Vatanen, Curtis Huttenhower, Ramnik J. Xavier

Reporting Summary

Nature Research wishes to improve the reproducibility of the work that we publish. This form provides structure for consistency and transparency in reporting. For further information on Nature Research policies, see Authors & Referees and the Editorial Policy Checklist.

Statistical parameters

When statistical analyses are reported, confirm that the following items are present in the relevant location (e.g. figure legend, table legend, main text, or Methods section).

n/a	Confirmed	
☐	☒	The exact sample size (n) for each experimental group/condition, given as a discrete number and unit of measurement
☐	☒	An indication of whether measurements were taken from distinct samples or whether the same sample was measured repeatedly
☐	☒	The statistical test(s) used AND whether they are one- or two-sided<br>*Only common tests should be described solely by name; describe more complex techniques in the Methods section.*
☐	☒	A description of all covariates tested
☐	☒	A description of any assumptions or corrections, such as tests of normality and adjustment for multiple comparisons
☐	☒	A full description of the statistics including central tendency (e.g. means) or other basic estimates (e.g. regression coefficient) AND variation (e.g. standard deviation) or associated estimates of uncertainty (e.g. confidence intervals)
☐	☒	For null hypothesis testing, the test statistic (e.g. F, t, r) with confidence intervals, effect sizes, degrees of freedom and P value noted<br>*Give P values as exact values whenever suitable.*
☒	☐	For Bayesian analysis, information on the choice of priors and Markov chain Monte Carlo settings
☐	☒	For hierarchical and complex designs, identification of the appropriate level for tests and full reporting of outcomes
☐	☒	Estimates of effect sizes (e.g. Cohen's d, Pearson's r), indicating how they were calculated
☐	☒	Clearly defined error bars<br>*State explicitly what error bars represent (e.g. SD, SE, CI)*

Our web collection on statistics for biologists may be useful.

Software and code

Policy information about availability of computer code

Data collection	No software was used for data collection.
Data analysis	Bowtie2 v2.2.3, Casava v1.8.2 (Illumina), cutadapt v1.9dev2, Trim Galore v0.2.8 (Babraham Bioinformatics), PRINSEQ v0.20.3, MetaPhlAn2 v2.6.0, HUMAnN2 v0.9.4, R v3.1.1, Python v2.7.1. Additional details are given in Methods.

For manuscripts utilizing custom algorithms or software that are central to the research but not yet described in published literature, software must be made available to editors/reviewers upon request. We strongly encourage code deposition in a community repository (e.g. GitHub). See the Nature Research guidelines for submitting code & software for further information.

Data

Policy information about availability of data

All manuscripts must include a data availability statement. This statement should provide the following information, where applicable:
- Accession codes, unique identifiers, or web links for publicly available datasets
- A list of figures that have associated raw data
- A description of any restrictions on data availability

TEDDY Microbiome 16S and WGS data that support the findings of this study are available in NCBI's database of Genotypes and Phenotypes (dbGaP) with the

primary accession code phs001443.v1, in accordance with the dbGaP controlled-access authorization process. Clinical metadata analyzed during the current study are available in the NIDDK Central Repository at https://www.niddkrepository.org/studies/teddy.

Field-specific reporting

Please select the best fit for your research. If you are not sure, read the appropriate sections before making your selection.

☒ Life sciences ☐ Behavioural & social sciences ☐ Ecological, evolutionary & environmental sciences

For a reference copy of the document with all sections, see nature.com/authors/policies/ReportingSummary-flat.pdf

Life sciences study design

All studies must disclose on these points even when the disclosure is negative.

Sample size	All stool samples with metagenomic data (N = 10,903 stool samples) from subjects in TEDDY islet autoimmunity and type 1 diabetes case-control cohorts are being analyzed for maximal power. This includes all subjects with islet autoimmunity or type 1 diabetes and their matched controls in TEDDY study as of May 31, 2012 (N =783 subjects).
Data exclusions	In T1D and IA case-control comparisons, all case-control pairs where the control later progressed to case status were removed (i.e. they progressed to IA or T1D).
Replication	For bacterial growth assays, the experiment was reproduced 3x with technical replicates in triplicate.
Randomization	Randomization was not used.
Blinding	No blinding was used, TEDDY is an observational follow-up study.

Reporting for specific materials, systems and methods

Materials & experimental systems

n/a	Involved in the study	
☐	☒	Unique biological materials
☒	☐	Antibodies
☒	☐	Eukaryotic cell lines
☒	☐	Palaeontology
☒	☐	Animals and other organisms
☐	☒	Human research participants

Methods

n/a	Involved in the study	
☒	☐	ChIP-seq
☒	☐	Flow cytometry
☒	☐	MRI-based neuroimaging

Unique biological materials

Policy information about availability of materials

Obtaining unique materials	The bacterial strains isolated for and used in this study will be provided to anyone who requests them.

Human research participants

Policy information about studies involving human research participants

Population characteristics	This study includes metagenomic profiles of stool samples from children collected monthly starting at three months of age to up to five years of age. The study population are 783 mostly white, non-hispanic children from six different clinical centers in the U.S. (Colorado, Georgia/Florida, and Washington) and Europe (Finland, Germany, and Sweden). The whole study population had a genetic predisposition for T1D or first-degree relative(s) with T1D.
Recruitment	Families with a newborn in participating clinical centers with HLA-conferred genetic predisposition for T1D or first-degree relative(s) with T1D were invited to join the study.
